# Phytochemical investigation and evaluation of anti-inflammatory and wound healing activities of *Plantago major* subsp. *intermedia* (Gilib.) Lange

**DOI:** 10.1186/s12906-026-05393-4

**Published:** 2026-04-29

**Authors:** Hilal Bacanak, Zeynep Dogan, Esra Küpeli, Akito Nagatsu, Iclal Saracoglu

**Affiliations:** 1 Faculty of Pharmacy, Department of Pharmacognosy, Hacettepe University, Ankara, 06100 Türkiye; 2 Faculty of Pharmacy, Department of Pharmacognosy, Tokat Gaziosmanpasa University, 60100 Tokat, Türkiye; 3 College of Pharmacy, Department of Pharmacognosy, Kinjo Gakuin University, Nagoya, 463‑8521 Japan; 4 Faculty of Pharmacy, Department of Pharmacognosy, Gazi University, Ankara, 06330 Türkiye; 5 Faculty of Pharmacy, Department of Pharmacognosy, Lokman Hekim University, Ankara, 06510 Türkiye

**Keywords:** Phytochemistry, Anti-inflammatory Activity, Wound Healing Activity, *Plantago major* subsp. *intermedia*

## Abstract

**Background:**

*Plantago major* subsp. *intermedia* has been traditionally used in Türkiye for the treatment of wounds, abscesses, constipation, pain, ulcers, hemorrhoids and gynecological diseases. In the present study, the phytochemical composition of *P. major* subsp. *intermedia* (Gilib.) Lange was investigated and its anti-inflammatory and wound healing effects were examined in light of existing experimental evidence from related *Plantago* species.

**Methods:**

MeOH, 80% EtOH and water extracts of the plant were prepared. In vivo anti-inflammatory effects of these extracts were investigated using an acetic acid-induced capillary permeability model and their wound healing effects were studied using linear incision and circular excision wound models. Isolation studies were performed on the 80% EtOH extract. Furthermore, in vitro studies were conducted on the 80% EtOH extract, the main fractions obtained from this extract and the some of the isolated pure compounds. In these in vitro studies, anti-inflammatory effects were investigated by measuring nitric oxide (NO), interleukin-6 (IL-6) and tumor necrosis factor-alpha (TNF-*α*) levels and wound healing effects were investigated using the scratch test.

**Results:**

A total of six compounds were isolated from *P. major* subsp. *intermedia*: Isotachioside (1), aucubin (2), 10-hydroxymajoroside (3), 10-acetoxymajoroside (4), martynoside (5) and acteoside (6). All tested compounds significantly inhibited NO production at concentrations of 25, 50 and 100 µM (12.68%-61.89%). Although in vivo results were not statistically significant, the 80% EtOH extract showed the highest efficacy in linear incision and circular excision wound models.

**Conclusions:**

The results of this study indicate the in vitro anti-inflammatory and wound healing activities of *P. major* subsp. *intermedia* and are consistent with previously reported biological activities of *P. major*.

**Supplementary Information:**

The online version contains supplementary material available at 10.1186/s12906-026-05393-4.

## Introduction

 Wound healing consists of four phases: hemostasis, inflammation, proliferation, and remodeling or maturation. The hemostasis phase begins with collagen release. Then, platelets clump together to form a clot, stopping bleeding. This clot acts as a wound matrix, stimulating cell migration. Inflammatory cells migrate to the wound site, releasing platelet-derived growth factors and cytokines [1]. In the inflammation phase, damaged cells release free radicals and reactive oxygen species (ROS). Vascular permeability increases, antimicrobial agents are released, and cells involved in tissue repair, such as keratinocytes and fibroblasts, are activated. In the proliferation phase, keratinocytes, fibroblasts, and endothelial cells proliferate, enabling angiogenesis, collagen synthesis, and re-epithelialization. This is followed by the remodeling/maturation phase, where newly formed tissues are organized, collagen fibers are aligned, and the wound area shrinks [2]. In this context, inflammation emerges as a crucial component of the wound healing process. Therefore, proper regulation of inflammation is essential for effective wound healing and tissue regeneration [3, 4].

Various compounds have been isolated from different *Plantago* species, including iridoids, flavonoids, triterpenes, steroids and their glycosides, as well as phenylethanoid glycosides and polysaccharides [5–7]. Among these, iridoid glycosides (e.g., aucubin, catalpol) and phenylethanoid glycosides (e.g., acteoside) are considered chemotaxonomically and pharmacologically important constituents of the genus *Plantago* [8, 9].

Numerous experimental studies have demonstrated that *P. major* exhibit anti-inflammatory and wound healing activities through the modulation of inflammatory mediators and tissue repair processes [10–13]. *P. major* has been shown to suppress key inflammatory mediators, including nitric oxide (NO), tumor necrosis factor-*α* (TNF-*α*) and interleukin-6 (IL-6), and to modulate cyclooxygenase (COX), lipoxygenase (LOX) and NF-κB (Nuclear Factor-kappa B) pathways [14–17]. Cream, gel formulations and nanoparticle-based preparations derived from *P. major* have demonstrated significant burn and wound healing effects [18–21]. Furthermore, *P. major* has demonstrated wound healing efficacy in animal models, including linear incision and excision wound assays, supporting their biological relevance in tissue repair [12, 22]. In addition to ethnomedicinal reports, experimental evidence from in vitro and in vivo studies has provided a scientific basis for evaluating *P. major* in validated pharmacological models.

Phytochemical and mechanistic studies indicate that iridoid glycosides, particularly aucubin, play a central role in these effects. Aucubin has been shown to inhibit pro-inflammatory cytokine production, downregulate iNOS (inducible nitric oxide synthase) and COX-2 expression and promote wound healing in both cellular and animal models [23–26]. Based on these experimentally validated mechanisms observed in *P. major* and its major constituents, the investigation of *P. major* subsp. *intermedia* using ethically approved in vivo and in vitro models is scientifically justified.

*P. major* subsp. *intermedia* (Plantaginaceae) is a perennial plant species and naturally distributed across much of Europe and in certain regions of Asia and Africa [27–29]. It is commonly known in Turkish as “siğil otu”, “damar otu”, “sinirli ot” and “bağa yaprağı”. In folk medicine in Türkiye, primarily the leaves of plant are used; however, the entire plant or the aerial parts may also be utilized for therapeutic purposes. The plant material is applied including crushed fresh or dried form, prepared as a decoction or infusion, or boiled with milk to make a poultice. Similar to *P. major*, *P. major* subsp. *intermedia* is traditionally used for wound healing, hemorrhoids, constipation, stomachache and inflammatory conditions [30–34]. However, unlike *P. major*, this subspecies is preferred in folk medicine for the treatment of knee pain, improperly healed bones and warts [35]. This difference in traditional use suggests that there may be differences in phytochemical content within the species. Our research group has previously conducted numerous studies on *Plantago* species and their bioactive components [36–38]. Our previous studies, particularly on *P. major* subsp. *major*, form the basis of our current research [39]. Furthermore, although there is a large amount of data in the literature regarding *Plantago major*, there is limited data on *Plantago major* subsp. *intermedia*. To this end, the phytochemical composition of the plant was investigated, and its biological activities were assessed using validated in vitro assays and ethically approved animal models.

## Materials and methods

### Chemicals and instruments

Solvents such as methanol (MeOH), ethanol (EtOH), chloroform and petroleum ether were supplied by Merck (Darmstadt, Germany). Aluminum-supported silica gel 60 plates (F_254_, 0.2 mm, Merck, Darmstadt, Germany) were used for thin-layer chromatography (TLC) analyses. Silica gel 60 (70–230 mesh, Merck, Darmstadt, Germany), silica gel-C_18_ (40–63 μm, Merck, Darmstadt, Germany) and polyamide (50–160 μm, Sigma-Aldrich, USA) were used as stationary phases in column chromatography. Deuterated MeOH (MeOH-d4, 99.8% D) used for NMR analyses was supplied by Isotec, Inc. (Miamisburg, OH, USA). Solvent removal was carried out with a Buchi R-210 vacuum evaporator (Flawil, Switzerland).

MTT, LPS, fetal bovine serum (FBS) and phosphate-buffered saline solutions were supplied by Sigma-Aldrich (MO, USA); penicillin-streptomycin and Dulbecco’s Modified Eagle Medium (DMEM) were supplied by Gibco Invitrogen Life Technologies (MA, USA). The IL-6 enzyme linked immunosorbent assay (ELISA) kit from FineTest (Wuhan, China) and the TNF-*α* ELISA kit from ELABScience (Wuhan, China) were purchased.

Medium-pressure liquid chromatography (MPLC) studies were conducted on a Buchi glass column (26 × 460 mm) containing LiChroprep C_18_ packing material, using a Buchi C-660 Fraction Collector, a Buchi C-615 Pump Manager and a Buchi C-605 Pump Module (Flawil, Switzerland). MeOH and distilled water were used as mobile phases. Eyela Micro Feeder MP-Σ pump (Rikakikai Co., Ltd., Tokyo, Japan), Develosil-RPAQUEOUS column (5 μm; 20 × 250 mm, Nomura Chemical, Aichi, Japan) and Jasco UV-2075 Plus UV-Vis detector (Tokyo, Japan) were used for preparative high-performance liquid chromatography (HPLC) analyses. ^1^H and ^13^C NMR spectra were obtained using a Jeol JMN-ECA600 (600 MHz) NMR instrument (Tokyo, Japan) and Jeol Delta software. During the scratch assay, wound closure was monitored microscopically using a Leica DM IL LED Fluo light microscope (Wetzlar, Germany). Microplate measurements were performed using the BioTek µQuant microplate reader (MQX200, VT, USA).

### Plant materials

Aerial parts of *P. major* subsp. *intermedia* were collected in June 2021 at the Hacettepe University Sıhhiye Campus, Altındağ, Ankara, Türkiye. Formal permission for plant collection was obtained from the Republic of Türkiye Ministry of Agriculture and Forestry, General Directorate of Nature Conservation and National Parks (Reference No: E-21264211-288.04-2307955). The study was conducted in accordance with national regulations. Taxonomic identification was conducted by Assistant Professor Dr. Z. Ceren Arıtuluk Aydın from the Department of Pharmaceutical Botany, Faculty of Pharmacy, Hacettepe University. A voucher specimen (HUEF 21023) was deposited in the Herbarium of the Faculty of the Pharmacy Hacettepe University (HUEF), Ankara, Türkiye. The plant material was shade dried and ground before extraction.

### Extraction and isolation

Experimental studies were conducted simultaneously to reveal the pharmacological potential of *P. major* subsp. *intermedia*. In this context, the systemic biological effects of various extracts prepared from the plant (MeOH, 80% EtOH and water) were tested in vivo. Simultaneously, the 80% EtOH extract was selected due to its TLC profile, and phytochemical isolation studies and in vitro experiments were carried out to determine its specific active components. Thus, we aimed to understand the systemic effect (in vivo) and mechanism of action of the plant.

For in vivo experiments, MeOH, 80% EtOH and water extracts were prepared from powdered *P. major* subsp. *intermedia*. Ten gram plant material was weighed for MeOH and 80% EtOH extracts. It was extracted three times at 40 °C for 8 h with the respective solvents (100 ml). The filtrates obtained were combined and the solvents were removed under vacuum in a rotavapor. MeOH (2.10 g; 21.98% w/w) and 80% EtOH (2.67 g; 26.65% w/w) extracts were obtained. For the water extract, 7.10 g of plant material was weighed and boiled with 525 ml of water for 30 min [40]. The obtained extracts were filtered and the water in the extract was removed under vacuum in a rotavapor. Thus, 1.33 g (18.73% w/w) of crude water extract was obtained. These three extracts were used for in vivo experiments.

When the TLC profiles of the MeOH, 80% EtOH and water extracts were compared, it was observed that the 80% EtOH extract had a more diverse phytochemical content. Therefore, 80% EtOH extract was preferred for phytochemical studies and in vitro biological activity studies. For this purpose, dried plant material (399.7 g) was extracted five times with 80% EtOH (3 L) at 40 °C for 8 h. The filtered and combined extracts were evaporated to dryness under reduced pressure at 40 °C. As a result, a total of 115.84 g (28.98% w/w) crude extract was obtained. To remove nonpolar compounds from the extract, the dry extract was dissolved in water and liquid-liquid extraction was performed with petroleum ether. The aqueous phase was evaporated on a rotavapor, obtaining 92.31 g of aqueous extract [Yield: 23.09 (a/a)]. 49.71 g of the aqueous extract was applied to the polyamide column. The elution process was started with 100% water, followed by 75:25%, 50:50%, 25:75% H_2_O: MeOH and 100% MeOH, respectively. Thus, five main fractions were obtained from the polyamide column.

Fr. A was subjected to liquid-liquid separation with *n*-butanol to remove excess sugars. Fr A was applied to MPLC. The column was set at flow rate 20 ml/min and temperature 25 °C. Frs. A_1−13_ were collected by running the column with a mobile phase of 5-100% MeOH. Fr. A_2_ was applied to silica gel column chromatography (SCC) with the solvent system CHCl_3_:MeOH (95:5–60:40). Compound **1** (2.9 mg) and compound **2** (9.2 mg) were obtained from the column. Fr. A_6_ was applied to SCC. Elution from the column was achieved with CHCl_3_:MeOH (95:5–30:70) mobile phase system and compound **3** (3.3 mg) was isolated. Fr. A_9_ was applied to SCC. Separation was performed on the column with the CHCl_3_:MeOH (100:0–65:35) mobile phase system. Compound **4** (2.9 mg) was obtained from the column. Fr. B was applied to SCC and eluted from the column with the CHCl_3_:MeOH (100:0–50:50) mobile phase system. Frs. B_1−13_ was obtained from the column. Fr. B_5_ was applied to VLC. Separation was performed on the H_2_O: MeOH (50:50 − 40:60) mobile phase system. Compound **5** (1.7 mg) was obtained from the column. Fr. B_11_ was applied to VLC and separation was performed with H_2_O: MeOH (65:35–50:50) mobile phase system (Frs. B_11a−h_). Compound **6** was obtained from the column. Frs. B_11f_ was applied to the HPLC system and analyzed. The column had a flow rate of 4 ml/min and a temperature of 25 °C. Compound **7** (2.5 mg) was purified with H_2_O: MeOH (55:45) solvent system.

### In vivo biological activity tests

#### Animals

Male Swiss albino mice (25–30 g) and male Sprague-Dawley rats (160–180 g) were obtained from the animal breeding laboratory of Kobay DHL A.Ş. The animals were housed under standard room conditions for three days and fed standard pellet chow and water ad libitum. Each group consisted of seven animals. All in vivo experiments were conducted in accordance with the European animal experimentation ethics guidelines and were approved by the Kobay Animal Experimentation Ethics Council (Project number: 786).

#### Preparation of test samples

For wound healing studies, the extracts (1%) were homogeneously mixed into an ointment base containing glyceryl stearate, 1,2-propylene glycol and liquid paraffin at a ratio of 3:6:1. 0.5 g dose of the prepared ointments was applied topically to the application site of linear incisional or circular excisional wound models created with a surgical blade. The vehicle group was treated with only the ointment base and the reference group was treated with Madecassol^®^ (Bayer) ointment containing 1% extract of *Centella asiatica*. No treatment was administered to the negative control group [41].

In anti-inflammatory studies, samples were prepared in a suspension containing distilled water and 0.5% sodium carboxymethyl cellulose (CMC) and administered orally. The control group was treated with only the vehicle solution while the reference group was treated with indomethacin (10 mg/kg) prepared in 0.5% CMC [42].

#### Wound healing activity

##### Linear incision wound model

The animals were anesthetized with 0.15 cc of Ketasol^®^ (Richterpharma). Their backs were shaved and disinfected with 70% alcohol. Two parallel 5 cm incisions, 1.5 cm apart on either side of the midline, were made with a sterile scalpel and each incision was closed with three surgical sutures placed 1 cm apart. Ointments containing the sample, reference ointment or carrier ointment were applied once daily for nine days. On the last day, sutures were removed and the wound tensile strength was measured with a tensiometer (Zwick/Roell Z0.5, Germany) [43–45].

##### Circular excision wound model

The animals were anesthetized with 0.01 cc of Ketasol^®^ (Richterpharma). The dorsal area of the animals was shaved. A circular excision wound was made between the shoulder blades with a biopsy punch (5 mm in diameter, Nopa Instruments, Germany). The wounds were left open and the test and reference ointments or carrier ointment were applied once daily until complete wound closure up to maximum 12 days. Wound healing was monitored daily with a camera (Fuji S20 Pro, Japan) and wound contraction was calculated using AutoCAD software [46, 47].

#### Anti-inflammatory activity

##### Acetic acid-induced capillary permeability

Anti-inflammatory effects were studied using an acetic acid-induced vascular permeability model according to a modified protocol of the Whittle method [48]. Test samples were administered orally to mice at a dose of 0.2 ml/20 g. After 30 min, 4% Evans blue solution (0.1 ml, Sigma, USA) was injected via the tail vein. Following this, 10 min later, 0.5% (v/v) acetic acid solution (0.4 ml) was administered intraperitoneally. After 20 min, the animals were euthanized by cervical dislocation. The internal organs were removed and washed with distilled water, the washings were transferred to 10 ml volumetric flasks containing 0.1 M NaOH and the volume was completed with distilled water. Absorbance was measured at 590 nm. The control group received distilled water and 0.5% CMC orally [42, 49].

The percentage inhibition of vascular permeability was calculated using the following formula:

$$\:Inhibition\:\left(\%\right)=\:\left[\frac{\left({A}_{control}-\:{A}_{treated}\right)}{{A}_{control}}\right]\times\:\:100$$

where $\:{A}_{control}$ represents the absorbance of the control group and $\:{A}_{treated}$ represents the absorbance of the treated group.

### In vitro biological activity tests

#### Cell culture

RAW 264.7 macrophages were obtained from Prof. Dr. Hasan Kırmızıbekmez at the Department of Pharmacognosy, Faculty of Pharmacy, Yeditepe University (Istanbul, Türkiye). The L929 fibroblast cell line was purchased from Biota Lab (Istanbul, Türkiye). Both cell lines were cultured in DMEM medium containing 10% FBS and 1% penicillin-streptomycin.

#### MTT assay for measuring cell viability

L929 and RAW 264.7 cells were cultured in DMEM medium supplemented with 10% FBS and 1% antibiotics in a humidified atmosphere with 5% CO_2_ at 37 °C. L929 cells were added at 10^5^ cells/mL and RAW 264.7 cells were added at 5 × 10^5^ cells/mL to 96-well plates and incubated for 24 h. After incubation, the medium was aspirated, 100 µL of test solutions at various concentrations were added and the cells were incubated for an additional 24 h. At the end of this period, 10 µL of MTT solution was added to the medium and incubated for 4 h. The medium was then discarded and replaced with 100 µL of DMSO. Absorbance values were measured at 577 nm with a reference wavelength of 655 nm using a microplate reader [50].

#### Anti-inflammatory activity by measuring NO and pro-inflammatory cytokines (TNF-*α* and IL-6)

The amount of nitrite produced in RAW 264.7 cells was determined using the Griess reagent [51]. Cells were plated at a density of 5 × 10^5^ cells/mL and incubated for 24 h. The medium was then removed and fresh medium containing various concentrations of test samples and LPS (0.2 µg/mL) was added and incubated for an additional 24 h. At the end of the incubation period, 100 µL of supernatant from each well was transferred to a new 96-well plate and an equal volume of Griess reagent [1% sulfanilamide in 5% phosphoric acid and 0.1% N-(1-naphthyl)ethylenediamine dihydrochloride in water] was added. Absorbance was measured at 577 nm to assess nitrite levels [38, 52]. IL-6 and TNF-*α* levels were determined from supernatants with ELISA kits in accordance with the manufacturer’s protocols [53, 54].

#### Wound healing activity by scratch wound assay

L929 cells were seeded in 96-well plates as 100 µL at a density of 10^5^ cells/mL and incubated for 24 h. At the end of incubation, an artificial wound was created by scratching the cell monolayer with a pipette tip and the medium was aspirated. 100 µl of test samples prepared at various concentrations were added to each well. The wound area was measured microscopically at 0. and 24. hours and the percentage of wound closure was calculated. DMEM containing 10% FBS was used as a positive control and DMEM without FBS was used as a negative control [55].

### Statistical analysis

Results are presented as mean ± standard error of mean (SEM) (*n* ≥ 3) from three or more independent replicates. To determine the differences between the groups, one-way ANOVA followed by Dunnett’s multiple comparison test was applied for in vitro studies and one-way ANOVA followed by the Student-Newman-Keuls post hoc test was applied for in vivo studies, using GraphPad Prism 10.0 software; *p* < 0.05 was accepted for statistical significance.

## Results

### Characterization of the isolated compounds

As a result of phytochemical studies on *P. major* subsp. *intermedia*, 6 compounds were isolated. Structural characterization of these compounds was done by 1D and 2D NMR techniques. The isolated compounds include aucubin (2) (C_15_H_22_O_9_; ESI-MS m/z 345.1 [M−H]^−^), 10-hydroxymajoroside (3), (C_17_H_24_O_11_; ESI-MS m/z 427.2 [M + Na]^+^) and 10-acetoxymajoroside (4) (C_19_H_26_O_12_; ESI-MS m/z 469.2 [M + Na]^+^) with an iridoid structure; martynoside (5) (C_31_H_40_O_15_; ESI-MS m/z 651.2 [M−H]^−^) and acteoside (6) (C_29_H_36_O_15_; ESI-MS m/z 623.2 [M−H]^−^) with a phenylethanoid glycoside structure. In addition, isotachioside (1) (C_13_H_18_O_8_; ESI-MS m/z 301.2 [M−H]^−^) a simple phenolic compound, was also obtained [56–60]. 1D (^1^H and ^13^C) and 2D (COSY, HMQC and HMBS) NMR data for the compounds are presented as Supplementery Material.

Isotachioside (1), martynoside (5) and acteoside (6) could not be included in biological activity studies because they were obtained in low amounts. Biological activity studies of other compounds [aucubin (2), 10-hydroxymajoroside (3) and 10-acetoxymajoroside (4)] were performed.

### In vivo biological activity test results

#### Linear incision wound model

The wound healing effects of MeOH, 80% EtOH and water extracts of *P. major* subsp. *intermedia* were evaluated using a linear incision wound model. 80% EtOH extract (15.2%) showed the highest effect. It was determined that MeOH extract (14.2%) was nearly as effective as 80% EtOH extract (Table 1).

**Table 1:** Effects of test and reference materials on wound contraction with linear incision model

Material	Extract type	Tensile strength ± S.E.M.	(Tensile strength %)
Vehicle	-	18.25 ± 3.37	-
Negative Control	-	19.73 ± 2.61	-
*Plantago major* subsp. *intermedia*	MeOH	15.67 ± 2.43	14.1
	80% EtOH	21.03 ± 2.14	15.2
	Water	18.51 ± 1.28	1.4
Madecassol^®^	-	24.49 ± 1.15	34.2**

Mean ± SEM; comparison with vehicle group and negative control

The % Tensile Strength values represent the relative increase calculated against the control groups

** *p* < 0.01

#### Circular excision wound model

To examine the wound healing potential, the percentage of wound closure was measured in vivo using a circular excision wound model. When the extracts were compared, the highest effect was observed with the 80% EtOH extract, which resulted in 23.4% wound closure on day 12. To improve clarity, Table 2 has been simplified to include only the percentage of contraction. The complete dataset is provided in Supplementary Material Table S6.

**Table 2:** Effects of test and reference materials on wound contraction with circular excision model

Material	Extract type	Wound area ± S.E.M. (Contraction %)			
		0	4	8	12
Vehicle	-		3.9	16.5	13.7
Negative Control	-	18.34 ± 2.19	16.92 ± 2.11	9.83 ± 1.62	4.80 ± 1.16
*Plantago major* subsp. *intermedia*	MeOH	nd	5.9	7.4	20.1
	80% EtOH	0.6	9.9	11.8	23.4
	Water	0.87	0.12	nd	nd
Madecassol^®^		nd	(30.0)*	(50.9)**	**(100.00)** ^***^

Mean ± SEM; comparison with vehicle group and negative control

*p < 0.05, ** *p* < 0.01, *** *p* < 0.001

#### Acetic acid-induced capillary permeability results

The anti-inflammatory potentials of MeOH, 80% EtOH and water extracts prepared from *P. major* subsp. *intermedia* aerial parts were evaluated using an acetic acid-induced capillary permeability model. The findings revealed that the MeOH extract provided the highest inhibition rate at 14.5%. (Table 3).

**Table 3:** Effects of test and reference materials on inflammation with acetic acid-induced capillary permeability model

Material	Extract type	Dose (mg/kg)	Evans blue concentration (µg/mL) ± SEM	Inhibition (%)
Control			10.91 ± 1.13	
* Plantago major* subsp. *intermedia*	MeOH	100	9.33 ± 0.94	14.5
	80% EtOH	100	10.38 ± 0.53	4.8
	Water	100	11.49 ± 0.65	-5.3
Indomethacin		10	5.01 ± 0.39	54.1***

Mean ± SEM; comparison with vehicle group and negative control

*** *p* < 0.001

### In vitro biological activity test results

#### MTT assay

**Fig. 1:**
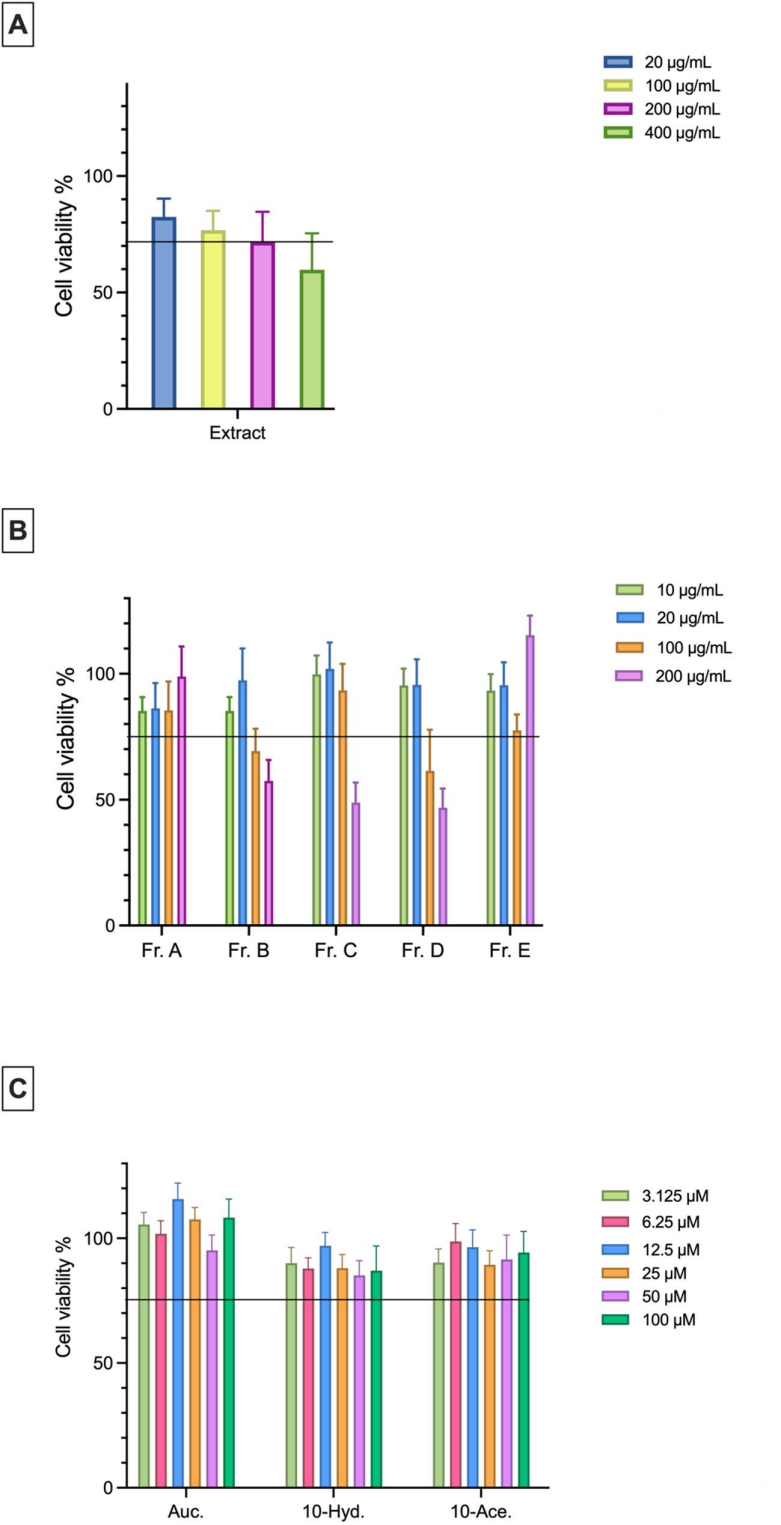
Cytotoxic effects of extract (**A**), fractions (**B**) and pure compounds (**C**) on cell viability in L929 cells (MTT test; mean ± SEM, *n* = 3) Auc: Aucubin, 10-Hyd: 10- Hydroxymajoroside, 10-Ace: 10-Acetoxymajoroside

**Fig. 2:**
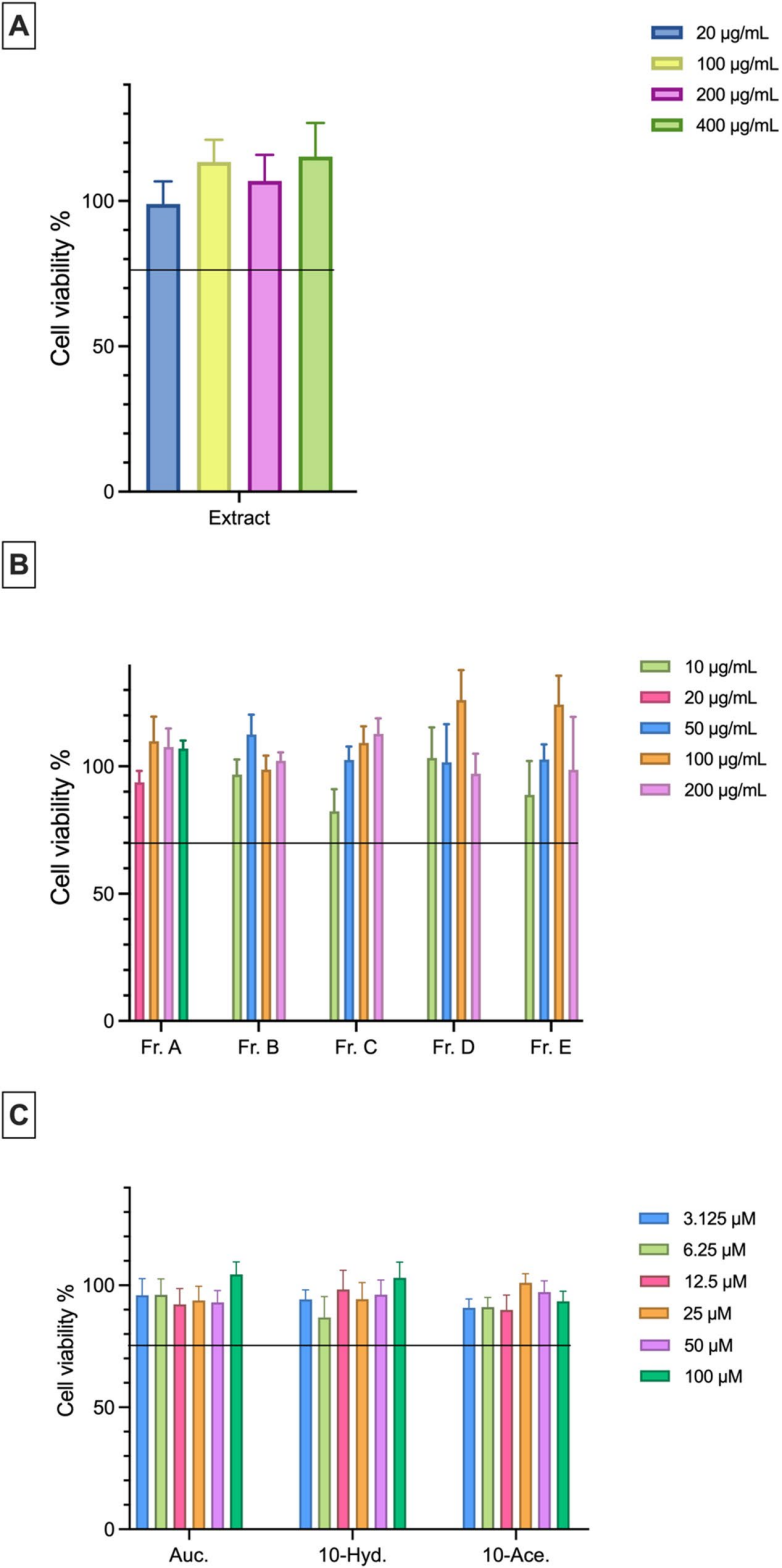
Cytotoxic effects of extract (**A**), fractions (**B**) and pure compounds (**C**) on cell viability in RAW 264.7 cells (MTT test; mean ± SEM, *n* = 3) Auc: Aucubin, 10-Hyd: 10- Hydroxymajoroside, 10-Ace: 10-Acetoxymajoroside

The concentrations of the samples that were non-toxic to cells were determined. For this purpose, cytotoxicity assays were performed using the MTT method on L929 and RAW 264.7 cell lines. Concentrations of 20, 100, 200 and 400 µg/mL were selected for the extract, while 10, 50, 100 and 200 µg/mL concentrations were used for the fractions. Pure compounds were prepared in six different concentrations ranging from 100 to 3.125 µM using two-fold serial dilution. In general, the results revealed that the samples did not exhibit any significant cytotoxic effects. However, the highest tested concentrations of the extract (59.75%; 400 µg/mL), Fr. B (57.41%; 200 µg/mL), Fr. C (48.86%; 200 µg/mL) and Fr. D (46.82%; 200 µg/mL) showed cytotoxic effects on L929 cells (Fig. 1). All samples exhibited cell viability in the range of 82.48%-126.1% in RAW 264.7 cells (Fig. 2). The 75% viability level is shown with a line in the graphs (Figs. 1 and 2) [61].

#### Effects on LPS-induced NO, IL-6 and TNF-*α* cytokine production

**Fig. 3:**
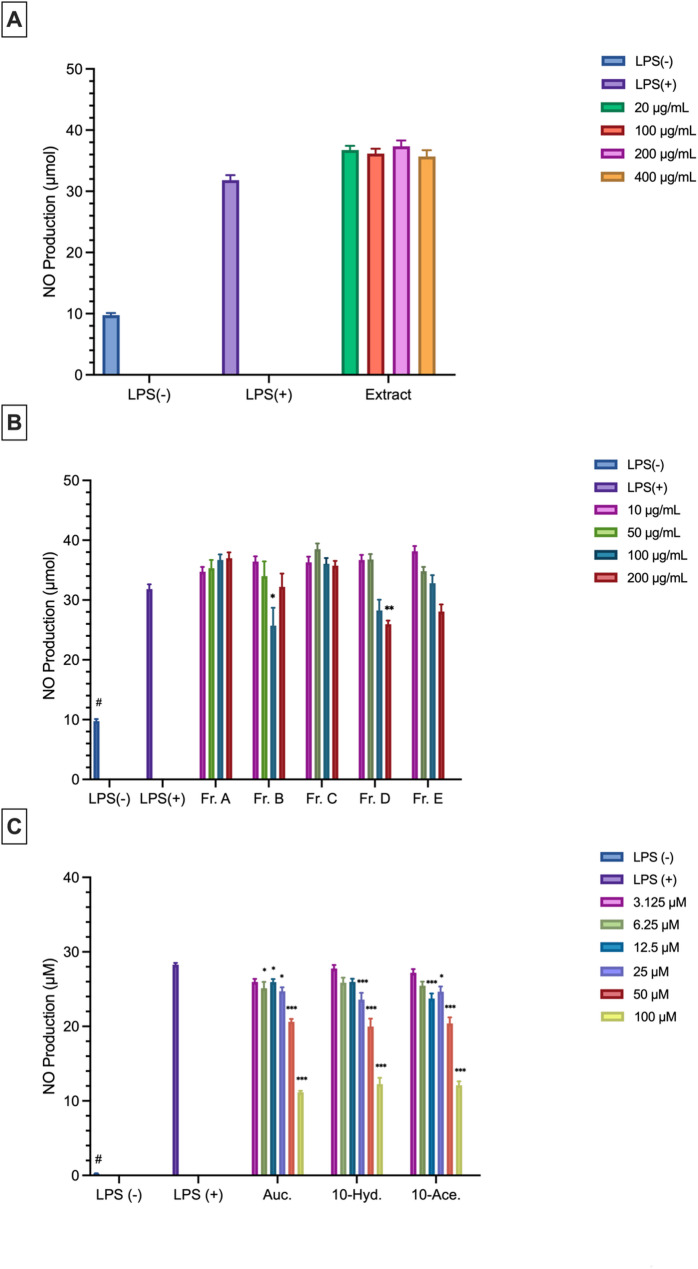
Anti-inflammatory effects of extract (**A**), fractions (**B**) and pure compounds (**C**) assessed via NO levels in LPS-induced RAW 264.7 cells (5 × 10^5^ cells/mL). Data are presented as mean ± SEM (*n* = 3). LPS (+), positive control; LPS (−), negative control. Statistical analysis was performed using one-way ANOVA followed by Dunnett’s multiple comparison test. *, **, and *** indicate statistically significant differences compared to the LPS (+) group ( *p* < 0.05, ** *p* < 0.01, *** *p* < 0.001). Auc: Aucubin, 10-Hyd: 10- Hydroxymajoroside, 10-Ace: 10-Acetoxymajoroside

**Fig. 4:**
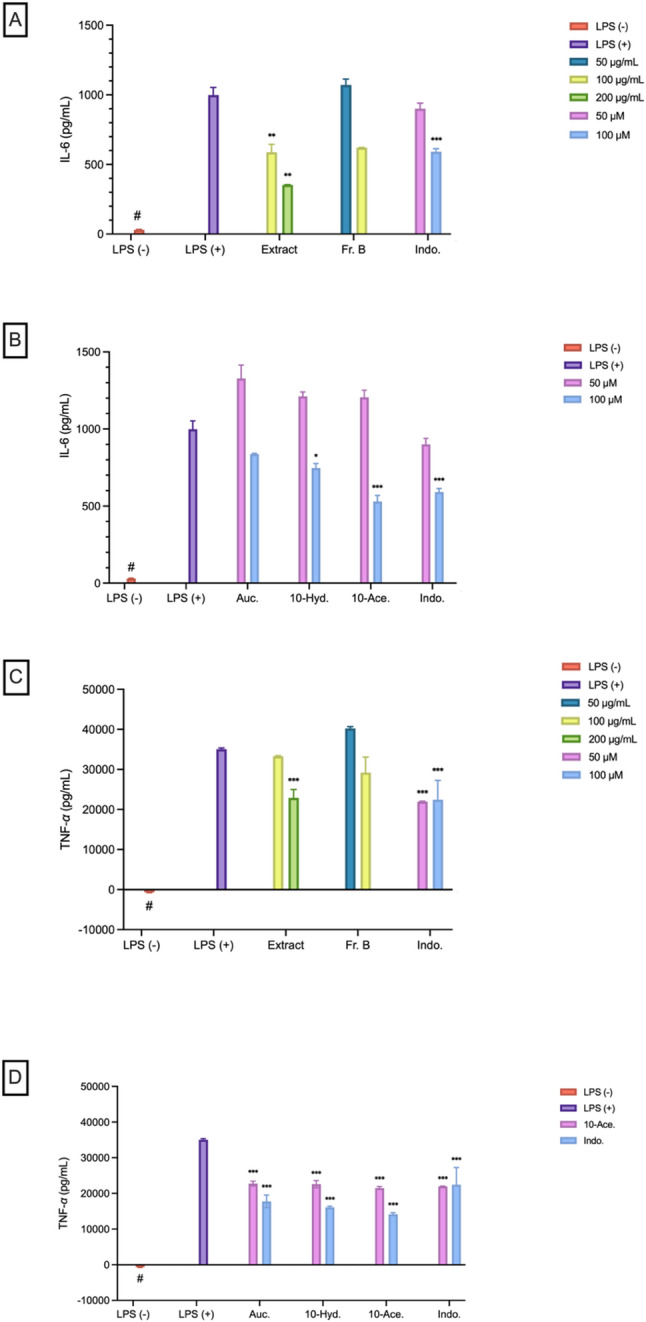
Anti-inflammatory effects of extract, Fr. B and pure compounds assessed via IL-6 extract and fractions (**A**); IL-6 pure compounds (**B**); TNF-a extract and fractions (**C**); IL-6 pure compounds (**D**) levels in LPS-induced RAW 264.7 cells (5×10^5^ cells/mL) Data are presented as mean ± SEM (n = 3). LPS (+), positive control; LPS (−), negative control. Statistical analysis was performed using one-way ANOVA followed by Dunnett’s multiple comparison test. *, *, and *** indicate statistically significant differences compared to the LPS (+) group ( *p* < 0.05, ** *p* < 0.01, *** *p* < 0.001). Auc: Aucubin, 10-Hyd: 10- Hydroxymajoroside, 10-Ace: 10-Acetoxymajoroside, Indo: Indomethacin

After LPS-induced stimulation in RAW 264.7 cells, the extract at 100 and 200 µg/mL concentrations significantly reduced IL-6 levels (42.47–66.63%) and the extract at 100 µg/mL concentration significantly reduced TNF-*α* levels (29.26%), compared to the LPS (+) group. But, it did not cause a significant decrease in NO levels at any tested concentration (Figs. 3A and 4A and C). In contrast, Fr. B (27.65% inhibition at 100 µg/mL) and Fr. D (26.61%; 200 µg/mL) markedly suppressed NO production (Fig. 3B). All compounds tested for activity significantly inhibited NO production at 25, 50 and 100 µM concentrations, with inhibition rates ranging from 12.68% to 61.89% (Fig. 3C). Additionally, aucubin (11.22%; 6.25 µM) and 10-acetoxymajoroside (16.22%; 12.5 µM) significantly reduced NO levels (Fig. 3C). 10-hydroxymajoroside and 10-acetoxymajoroside significantly decreased IL-6 levels at 100 µM (26.08–48.44%) (Fig. 4B). Similarly, aucubin, 10-hydroxymajoroside and 10-acetoxymajoroside provided significant reductions in TNF-*α* levels at 50 and 100 µM concentrations (29.58–50.30%) (Fig. 4D).

#### Scratch assay

In vitro wound healing activities of the extract, fractions and pure compounds were investigated using the scratch test (Tables 4, 5 and 6). According to cytotoxic activity results, concentration ranges of 20–200 µg/mL were selected for extract and 10–100 µg/mL for fractions. Pure compounds were prepared by two-fold serial dilution method and examined at six different concentrations ranging from 100 to 3.125 µM. In the scratch assay, in vitro wound healing effect of the samples was statistically demonstrated by comparing them with the FBS (-) control group. Among the fractions, Fr. A showed the highest effect with 67.74–91.87% wound closure in the 10–100 µg/mL range (Table 5). Fr. D provided 64.17–88.64% wound closure at concentrations of 10 and 50 µg/mL (Table 5). Among the isolated compounds, the highest wound closure was observed with aucubin (69.33%; 6.25 µM) (Table 6). Furthermore, 10-acetoxymajoroside at 25 and 50 µM (58.25–64.65%) and 10-hydroxymajoroside at 12.5 and 6.25 µM (60.81–61.45%) showed the highest wound healing activities (Table 6).

**Table 4:** Wound healing promoting effects of extract in the scratch wound model induced in L929 fibroblasts

Materials	Closure % (Statistical Mean ± S.E.M.)			
	20 µg/mL	50 µg/mL	100 µg/mL	200 µg/mL
Extract	56.84 ± 5.24 ***	58.56 ± 4.91 ***	61.10 ± 1.54 ***	nd
FBS (+)	90.04 ± 5.08 #			
FBS (-)	5.62 ± 1.75			

Mean ± SEM, *n* = 3; FBS (+)/FBS (–) control; 1 × 10⁵ cells/mL

nd not determined due to cytotoxicity at this concentration (cell viability < 75%)

*** *p* < 0.001

**Table 5:** Wound healing promoting effects of fractions in the scratch wound model induced in L929 fibroblasts

Materials	Closure % (Statistical Mean ± S.E.M.)			
	10 µg/mL	20 µg/mL	50 µg/mL	100 µg/mL
Fr. A	91.87 ± 5.28 ***	87.79 ± 6.45 ***	67.74 ± 3.01 ***	29.72 ± 0.98 **
Fr. B	49.47 ± 4.28 ***	30.49 ± 1.41 **	nd	nd
Fr. C	33.63 ± 3.79 **	20.67 ± 2.80	17.15 ± 1.90	nd
Fr. D	88.64 ± 6.90 ***	64.17 ± 5.45 ***	nd	nd
Fr. E	22.68 ± 0.92	43.64 ± 1.27 ***	20.71 ± 1.93	24.81 ± 0.78 *
FBS (+)	90.04 ± 5.08 #			
FBS (-)	5.62 ± 1.75			

Mean ± SEM, *n* = 3; FBS (+)/FBS (–) control; 1 × 10⁵ cells/mL

*nd* Not determined due to cytotoxicity at this concentration (cell viability < 75%)

* *p* < 0.05, ** *p* < 0.01, *** *p* < 0.001

**Table 6:** Wound healing promoting effects of pure compounds in the scratch wound model induced in L929 fibroblasts

Materials	Closure % (Statistical Mean ± S.E.M.)					
	3.125 µM	6.25 µM	12.5 µM	25 µM	50 µM	100 µM
Auc.	11.21 ± 0.99	69.33 ± 0.97 ^***^	6.02 ± 0.33	2.35 ± 0.332	19.24 ± 1.12 ^***^	5.24 ± 0.34 ^***^
10-Hyd.	36.19 ± 1.89 ^***^	61.45 ± 2.28 ^***^	60.81 ± 1.72 ^***^	33.49 ± 1.44 ^***^	45.53 ± 1.55 ^***^	20.17 ± 1.12 ^***^
10-Ace.	11.72 ± 1.17	1.98 ± 0.62	52.47 ± 0.79 ^***^	58.25 ± 0.82 ^***^	64.65 ± 1.91 ^***^	45.74 ± 1.55
FBS (+)	96.84 ± 2.02					
FBS (-)	4.99 ± 1.67					

Mean ± SEM, *n* = 3; FBS (+)/FBS (–) control

*Auc* Aucubin, *10-Hyd* 10- Hydroxymajoroside, *10-Ace* 10-Acetoxymajoroside

*** *p* < 0.001, (1 × 10^5^ cells/mL)

## Discussion

This study aims to investigate the phytochemical composition of *P. major* subsp. *intermedia*, as well as its anti-inflammatory, wound-healing, and antioxidant activities.

The anti-inflammatory activities of the MeOH, 80% EtOH and water extracts were assessed in vivo using the acetic acid-induced capillary permeability assay, whereas their wound healing potentials were tested through linear incision and circular excision models. Overall, none of the three extracts produced statistically significant outcomes in vivo assays. Nevertheless, the MeOH extract demonstrated the strongest effect in the capillary permeability assay, while the 80% EtOH extract exhibited the most pronounced activity in the linear incision and circular excision wound models. In the literature, MeOH and EtOH extracts of various *Plantago* species have been reported to exhibit strong anti-inflammatory and wound healing activities [22, 62, 63]. Although our in vivo results did not reveal any statistically significant effects, the relatively higher activities of MeOH and 80% EtOH extracts were found to be consistent with the literature. Similar anti-inflammatory effects of *P. major* extracts prepared with alcoholic solvents have been reported in previous in vivo study [15]. Although MeOH and 80% EtOH are similar in terms of polarity, extraction efficiency and selectivity are affected not only by polarity but also by solvent composition. The presence of water in 80% EtOH can increase the solubility of glycosidic compounds, particularly iridoid glycosides and phenylethanoids, which are abundant in plants and readily soluble in water due to their sugar moieties [64]. Consequently, 80% EtOH may have enabled more efficient extraction of these compounds compared to MeOH. This is thought to have resulted in the 80% EtOH extract having higher biological activity than the MeOH extract. Isotachyside (1), aucubin (2), 10-hydroxymajorside (3), 10-acetoxymajorside (4), martynoside (5) and acteoside (6) were obtained from the 80% EtOH extract of *P. major* subsp. *intermedia* [56–60]. According to the available information in the literature, isotachioside was isolated from a *Plantago* species for the first time. To date, 10-acetoxymajoroside has been reported to have been isolated from nature only once, from *P. major* [57]. Also, to our knowledge 10-hydroxymajoroside has been previously isolated from nature only three times, from *P. cornuti* and *P. major* subsp. *intermedia* [57, 58, 65]. The cytotoxicity of 80% EtOH extract, fractions and pure compounds was investigated in L929 and RAW 264.7 cell lines. In L929 cells, extract (59.75%; 400 µg/mL), Fr. B (57.41%; 200 µg/mL), Fr. C (48.86%; 200 µg/mL) and Fr. D (46.82%; 200 µg/mL) showed lower cell viability at some concentrations compared to other samples. Except for the concentrations specified, none of the samples showed cytotoxic effects. Although these values are not considered highly cytotoxic, they were interpreted as indicating a moderate reduction in cell viability. These findings are consistent with previous studies reporting low cytotoxicity of *Plantago* species and their major constituents, such as iridoid glycosides and phenolic compounds, in various cell lines [14, 38, 61, 66]. The absence of significant cytotoxicity supports the relative pharmacological safety of the tested samples under the experimental conditions applied. After determining non-cytotoxic concentrations, the effects of the extract, fractions and pure compounds on inflammation were assessed by measuring NO, IL-6 and TNF-*α* levels in LPS-induced RAW 264.7 cells. A previous study demonstrated that a 70% EtOH extract of *P. major* leaves decreased PGE_2_ (Prostoglandin E_2_) and TXA_2_ (Thromboxane A_2_) levels, an effect attributed to the downregulation of PLA_2_ (Phospholipase A_2_) enzyme expression [14]. In another study, the anti-inflammatory effects of water, EtOH and water+EtOH combination extracts obtained from *P. major* were investigated by measuring the translocation of NF-κB between the cytoplasm and the nucleus. It was determined that all three extracts acted via this pathway [16]. In our study, it was observed that the *P. major* subsp. *intermedia* extract decreased NO levels by 19% at a concentration of 100 µg/mL. Furthermore, it was observed to significantly decreased IL-6 levels at concentrations of 100 and 200 µg/mL, and TNF-*α* levels at a concentration of 200 µg/mL. NO, IL-6 and TNF-*α* cytokines are known to be associated with the activation of the NF-κB pathway [67]. When our data are evaluated together with the literature, it is thought that the effect of our extract on NO, IL-6, and TNF-*α* may occur through the NF-κB pathway.

In our study, Fr. B (27.65%) at 100 µg/mL and Fr. D (26.61%) at 200 µg/mL significantly reduced NO levels. A review of the literature indicates that phenolic compounds have anti-inflammatory effects [68, 69]. Based on TLC profiles and phytochemical analyses, these effects are thought to be attributed to the phenolic compound content of the fractions. Three isolated compounds (11.22–61.89%) tested in vitro significantly inhibited NO levels at various concentrations. 10-hydroxymajoroside and 10-acetoxymajoroside significantly reduced IL-6 levels at 100 µM, while aucubin, 10-hydroxymajoroside and 10-acetoxymajoroside decreased TNF-*α* levels at 50 µM and 100 µM. The anti-inflammatory effects of aucubin, 10-hydroxymajoroside and 10-acetoxymajoroside are thought to be due to their iridoid glycoside structures [70]. In a previous study on 10-hydroxymajoroside, its ABTS radical scavenging activity was investigated and at a concentration of 100 µg/mL, the compound showed 66% inhibition against the ABTS radical [65]. When evaluated together with our findings, it was thought that the anti-inflammatory effect of the compound may be related to its antioxidant activity.

In a study, aucubin was reported to prevent inflammation and matrix degradation in IL-1*β*-induced articular chondrocytes by suppressing MMP-3, MMP-9, MMP-13, COX-2 and iNOS expression, as well as by inhibiting NF-κB activation [71]. A study investigated the anti-inflammatory mechanism of aucubin on rheumatoid arthritis using in vitro and in vivo experiments. In vitro studies used HFLS-RA (Human Fibroblast-Like Synoviocytes - Rheumatoid Arthritis), RAW 264.7 and osteoblast progenitor cells obtained from rheumatoid arthritis patients. In vivo, a collagen-induced arthritis rat model was applied. The study results showed that aucubin reduced the expression of inflammation-related cytokines (IL-6, TNF-*α *and IL-1*β*) and MMPs. It was determined that it suppressed inflammation by inhibiting the NF-κB signaling pathway [72]. Another study investigated the anti-inflammatory effect of aucubin in glucocorticoid-induced femoral head osteonecrosis. Aucubin was shown to reduce levels of cytokines (IL-6, TNF-*α *and IL-1*β*) and suppress inflammation by inhibiting the TLR4/NF-κB (Toll-Like Receptor 4) signaling pathway [73]. In a study the effect of aucubin on inflammatory bowel disease. It was observed that aucubin maintained intestinal barrier integrity in a DSS-induced colitis model. Furthermore, it was concluded that by suppressing the MyD88/NF-κB signaling pathways, it significantly reduced the release of pro-inflammatory cytokines such as IL-6, TNF-*α *and IL-1*β* [74]. Another study showed that aucubin reduced NO and PGE_2_ production in LPS-stimulated microglia cells. Aucubin also suppressed iNOS and COX-2 at both protein and mRNA levels. It has also been observed to inhibit the activation of NF-κB, PI3K/Akt and MAPK signaling pathways [23]. When these findings are evaluated together with our results, it suggests that the anti-inflammatory effect of aucubin may be partly related to the suppression of the NF-κB, PI3K/Akt and MAPK signaling pathway.

As a result of our study, in the scratch assay, among all fractions, Fr. A showed the highest wound closure effect (67.74–91.87%). Additionally, the effect of Fr. D on wound closure (64.17–88.64%) was observed to be comparable to that of Fr. A. It was concluded that the effect of Fr. A may be attributed to its content of iridoids while the effect of Fr. D may be due to its phenolic compounds. Among the isolated compounds, aucubin (69.33%; 6.25 µM) exhibited the highest activity, suggesting that the strong wound healing effect observed with Fr. A could be derived from its aucubin content.

The healing effect of aucubin on wounds was evaluated in a diabetic wound model using hyperglycemic rats. Rats were divided into 4 groups: P1 (aucubin gel 20 µg), P2 (aucubin gel 40 µg), K1 (Bioplacenton^®^) and K2 (gel base/negative control). The results revealed that aucubin gel (20 and 40 µg) showed 100% wound closure effect compared to the negative control group (83%). Additionally, aucubin gel significantly shortened the wound healing time, reducing it from 24.4 days (negative control) to 11.7 days [25].

Furthermore, in the current study, 10-acetoxymajoroside (58.25–64.65%) and 10-hydroxymajoroside (60.81–61.45%) also showed significant wound healing activities. It was concluded that the high wound healing effects of the compounds (**3** and **4**) may be attributed to their iridoid glycoside nature and structural similarity to aucubin.

The wound-healing effect of a hydrogel prepared from *P. major* extract was measured using the scratch test. As a result, it was observed that the hydrogel stimulated fibroblast migration, leading to wound closure [20]. MeOH extract was prepared from *P. major* leaves by maceration method. In vivo wound healing effect of the extract was investigated in an excision wound model experiment. In order to elucidate the mechanism of action of the extract in wound healing, its anti-inflammatory effect was investigated in LPS-stimulated RAW cells using NO quantification test, fibroblast proliferation assay and migration assay in high glucose medium. It was determined that the extract significantly increased wound closure (% wound closure: 98 ± 1) on day 14. In the NO quantification test, it was tested at concentrations of 62.5, 125 and 250 µg/mL and it significantly reduced NO at all concentrations. It was observed that the extract increased fibroblast proliferation in the concentration range of 31–500 µg/mL. In the migration test in high glucose medium, the extract was tested at concentrations of 250, 500 and 1000 µg/mL. It was concluded that it did not increase cell migration at any concentration [22].

A systematic review evaluated the effectiveness of topical application of *P. major* on wound healing in animal models. Data were included in the study between January 2006 and March 2020. Databases were searched in the Virtual Health Library, Public/Publisher MEDLINE, Scopus, Web of Science, Embase, Cumulative Index of Nursing and Allied Health Literature and CAB Direct. Of the 176 publications searched, only four met the inclusion criteria (20–100 animals). Wound healing time was found to be between 17 and 21 days. Wound closure was most pronounced at 10%, 20% and 50% concentrations compared to the control group [75]. Since the reference drug we used in our study provided complete closure on the 12th day, the effects of the extracts were evaluated up to the 12th day at most. These literature findings are coherent with the scratch analysis data obtained in our study on *P. major* subsp. *intermedia* extract. In a previous study, the anti-inflammatory effects of the MeOH extract of *P. subulata* and its derivatives, acteoside and isoacteoside, were investigated. NO, PGE_2_, and TNF-*α* levels were measured in LPS-stimulated RAW 264.7 cells. In the NO quantification assay, the extract was significantly effective at concentrations of 100, 200 and 400 µg/mL; while acteoside and isoacteoside were significantly effective at concentrations of 1 and 50 µg/mL (*p* < 0.05). The extract, acteoside, and isoacteoside reduced PGE_2_ and TNF-*α* cytokine levels [38].

In light of all these findings, the anti-inflammatory and wound healing potential of *P. major* subsp. *intermedia* has been supported by experimental evidence obtained from phytochemical, in vitro and in vivo studies. A detailed phytochemical analysis of the plant was conducted and comprehensive biological activity studies were performed on its extract, fractions and isolated compounds.

## Conclusions

This study presents experimental findings on the anti-inflammatory and wound-healing biological activity of *P. major* subsp. *intermedia*. The results are particularly supported by in vitro activities and chemical composition. While in vivo results were not statistically significant, they provided information about the effects of extracts prepared with different solvents. Future investigations are suggested to further elucidate the mechanisms of action of the isolated compounds and to confirm their therapeutic potential through preclinical and clinical evaluations.

## Supplementary Information

Supplementary Material 1.

## Data Availability

All data supporting the findings of this study are available within the article and its Supplementary Information files. The corresponding author provides the datasets and analysis on reasonable request.

## Abbreviations

CMC: Carboxymethyl Cellulose
COSY: Correlation Spectroscopy
COX: Cyclooxygenase
DMEM: Dulbecco’s Modified Eagle Medium
DMSO: Dimethyl Sulfoxide
ELISA: Enzyme Linked Immunosorbent Assay
ESI-MS: Electrospray Ionization Mass Spectrometry
EtOH: Ethanol
FBS: Fetal Bovine Serum
HFLS-RA: Human Fibroblast-Like Synoviocytes - Rheumatoid Arthritis
HMBC: Heteronuclear Multiple-Bond Correlation
HMQC: Heteronuclear Multiple-Quantum Coherence
HUEF: Herbarium of the Faculty of the Pharmacy Hacettepe University
HPLC: High Performance Liquid Chromatography
IC_50_: Inhibitory Concentration 50%
IL-1*β*: Interleukin-1 beta
IL-6: Interleukin-6
iNOS: Inducible Nitric Oxide Synthase
LOX: Lipoxygenase
LPS: Lipopolysaccharides
MAPk: Mitogen Activated Protein Kinase
MeOH: Methanol
MPLC: Medium-pressure liquid chromatography
MPP: Matrix Metalloproteinase
MTT: 3-(4,5-dimethylthiazol-2-yl)-2,5-diphenyl tetrazolium bromide
NF-κB: Nuclear Factor-kappa B
NMR: Nuclear Magnetic Resonance
NO: Nitric oxide
PGE_2_: Prostaglandin E_2_
PI3K/Akt: Phosphoinositide 3-kinase/Protein Kinase B
PLA_2_: Phospholipase A_2_
SCC: Silica Gel Column Chromatography
SEM: Standard Error of Mean
TLC: Thin Layer Chromatography
TLR4: Toll-Like Receptor 4
TXA E_2_: Thromboxane A_2_
TNF-*α*: Tumor Necrosis Factor alpha
