# Supplementary material for: Phytochemical investigation and evaluation of anti-inflammatory and wound healing activities of Plantago major subsp. intermedia (Gilib.) Lange
Source: BMC Complement Med Ther. 2026 Apr 29;26:212. doi: 10.1186/s12906-026-05393-4 (PMC13277164; doi:10.1186/s12906-026-05393-4)
Supplement: Supplementary file 1 — Supplementary Material 1. [file 12906_2026_5393_MOESM1_ESM.docx]

**Phytochemical Investigation and Evaluation of Anti-Inflammatory and Wound Healing Activities of *Plantago major* subsp*. intermedia* (Gilib.) Lange**

Hilal Bacanak ^1,2,3*^, Zeynep Dogan^1^, Esra Küpeli^4^, Akito Nagatsu^3^, Iclal Saracoglu^1,5^

*^1^ Hacettepe University, Faculty of Pharmacy, Department of Pharmacognosy, 06100 Ankara, Türkiye*

*^2^ Tokat Gaziosmanpasa University, Faculty of Pharmacy, Department of Pharmacognosy, 60100 Tokat, Türkiye*

*^3^ Kinjo Gakuin University, College of Pharmacy, Department of Pharmacognosy, 463‑8521 Nagoya, Japan*

*^4^ Gazi University, Faculty of Pharmacy, Department of Pharmacognosy, 06330 Ankara, Türkiye*

*^5^ Lokman Hekim University, Faculty of Pharmacy, Department of Pharmacognosy, 06510 Ankara, Türkiye*

**Table of Contents**

|  |  | **Page** |
| --- | --- | --- |
| **Figure S1** | Chemical structure of compounds isolated from *Plantago major* subsp. *intermedia* | **1** |
| **Table S1** | ^1^H and ^13^C NMR (CD_3_OD) data: chemical shift (δ, ppm) and coupling constant (*J*, Hz) for isotachioside (**1**). | **2** |
| **Table S2** | ^13^C (CD_3_OD) data: aucubin (**2**), 10-hydroxymajoroside (**3**) and 10-acetoxymajoroside (**4**). | **3** |
| **Table S3** | ^1^H NMR (CD_3_OD) data: chemical shift (δ, ppm) and coupling constant (*J*, Hz) for aucubin (**2**), 10-hydroxymajoroside (**3**) and 10-acetoxymajoroside (**4**). | **4** |
| **Table S4** | ^13^C NMR (CD_3_OD) data: acteoside (**6**) | **5** |
| **Table S5** | ^1^H NMR (CD_3_OD) data: chemical shift (δ, ppm) and coupling constant (*J*, Hz) for martynoside (**5**) and acteoside (**6**) | **6** |

**
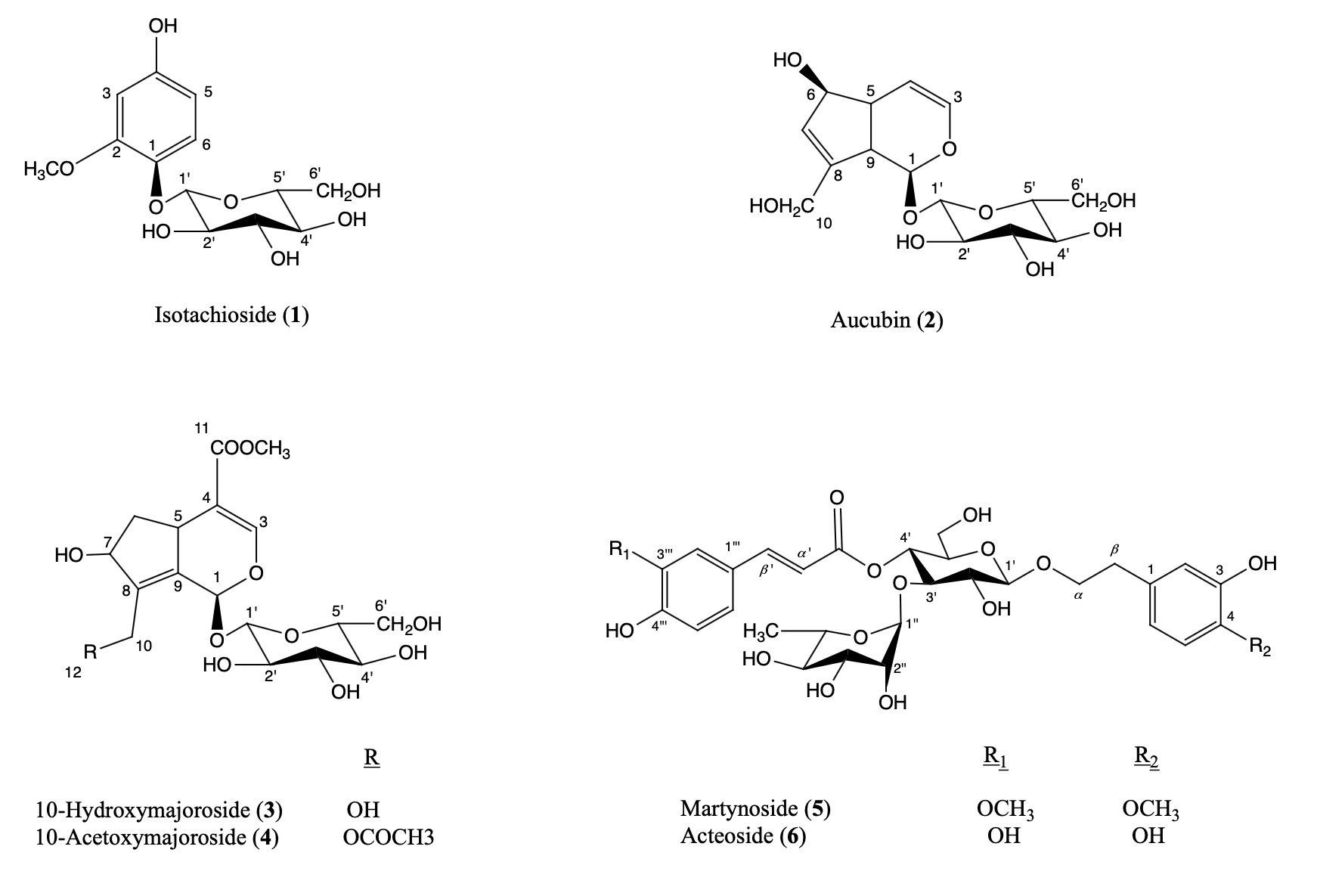
**

**Figure S1.** Chemical structure of compounds isolated from *Plantago major* subsp. *intermedia*

**Table S1.** ^1^H and ^13^C NMR (CD_3_OD) data: chemical shift (δ, ppm) and coupling constant (*J*, Hz) for isotachioside (**1**).

| **C/H** | **δ_c_** ppm | **δ_H_** ppm (mult., int., *J,* Hz) |
| --- | --- | --- |
| Aglycon | | |
| 1 | 141.3 | - |
| 2 | 152.3 | - |
| 3 | 102.1 | 6.46 (1H, d, 2.4 Hz) |
| 4 | 155.2 | - |
| 5 | 107.9 | 6.29 (1H, dd, 11.4 / 3.0 Hz) |
| 6 | 120.8 | 7.01 (1H, d, 8.4 Hz) |
| OCH_3_ | 56.8 | 3.80 (3H, s) |
| Glucose | | |
| 1' | 104.6 | 4.69 (1H, d, 7.2 Hz) |
| 2' | 75.4 | 3.32-3.44 † (1H) |
| 3' | 78.4 | 3.32-3.44 † (1H) |
| 4' | 71.7 | 3.32-3.44 † (1H) |
| 5' | 78.2 | 3.42 (1H, d, 4.2 Hz) |
| 6' | 62.9 | 3.89 (1H, dd, 2.4 / 12.0 Hz) |
|  |  | 3.68 (1H, dd, 5.4 / 12.0 Hz) |

†: The *J* value could not be calculated due to interference

**
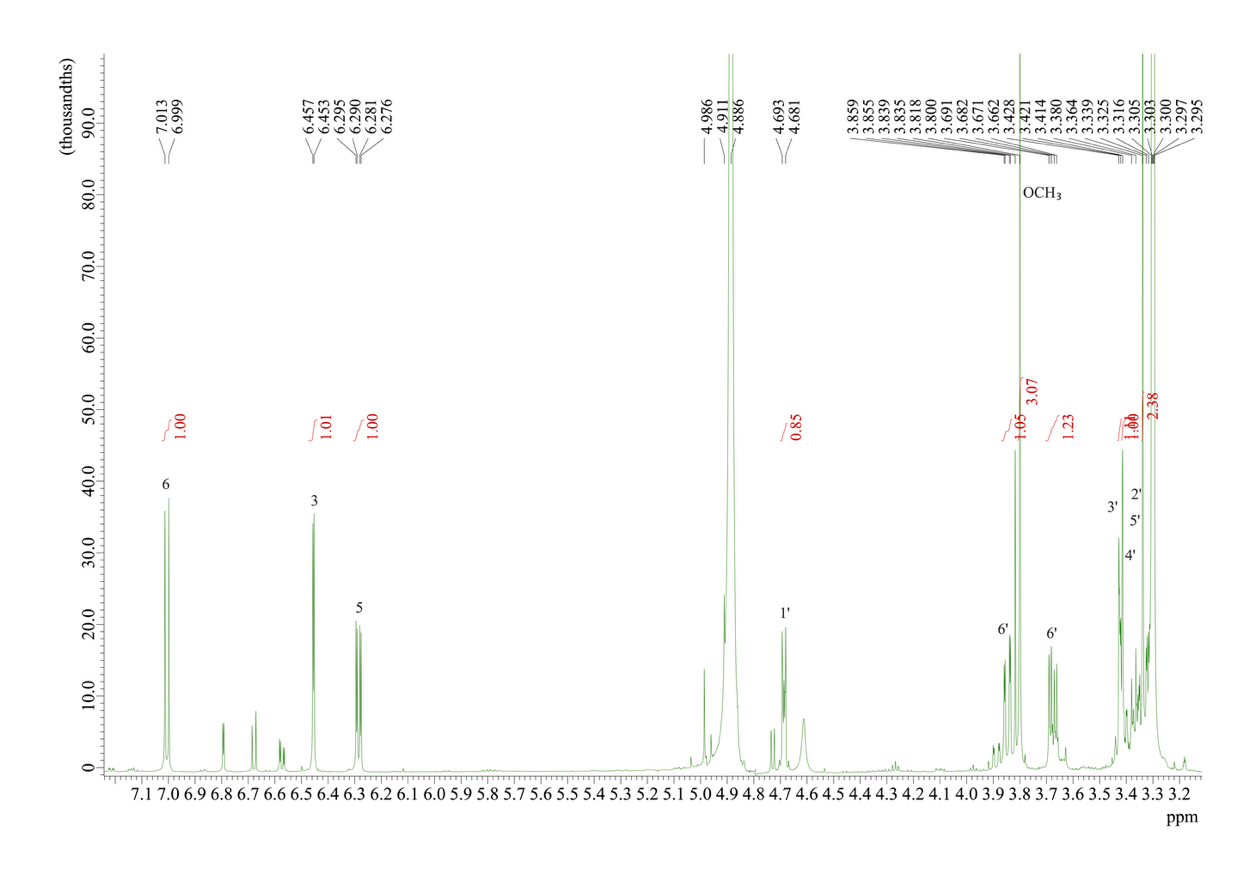
**

**Figure S2.** ^1^H-NMR spectrum of isotachioside (CD_3_OD; 600 MHz).

**
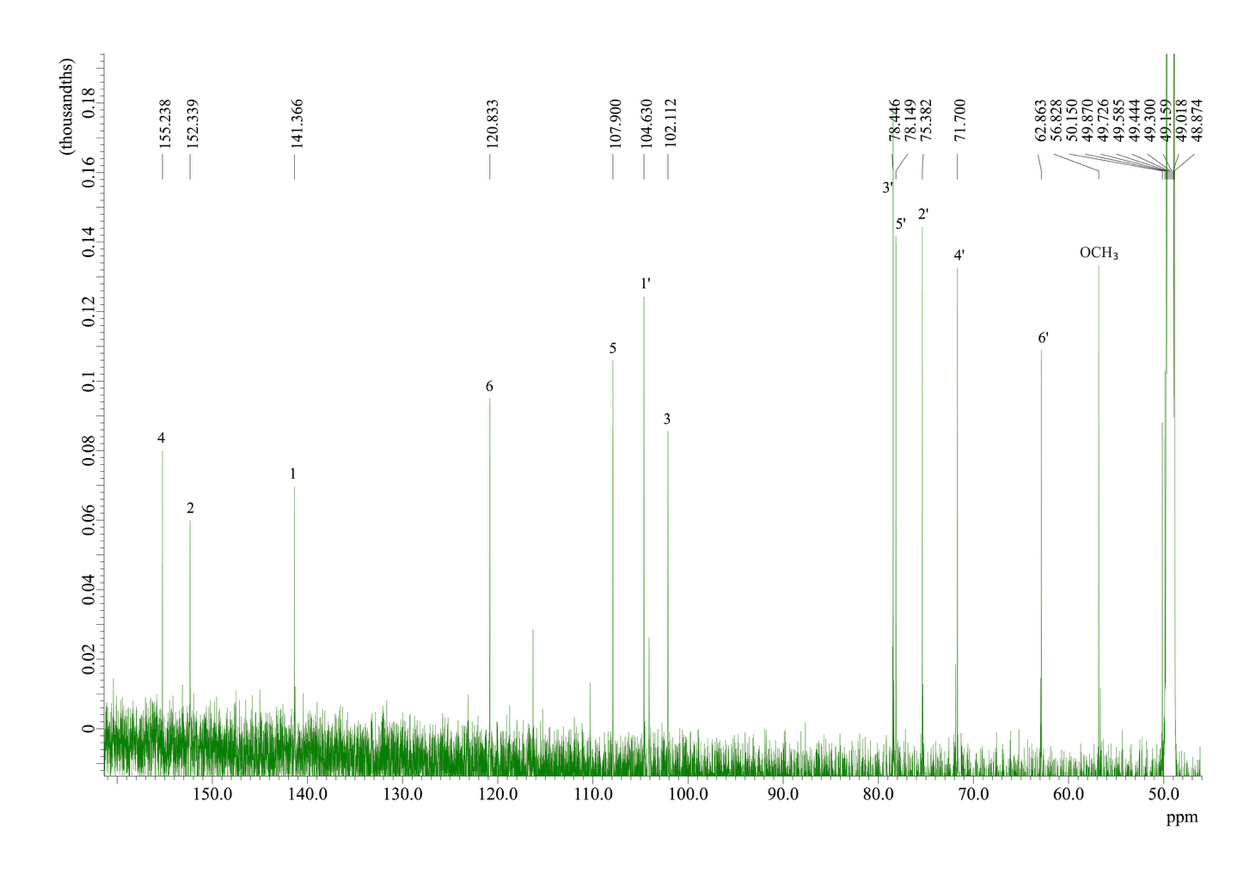
**

**Figure S3.** ^13^C-NMR spectrum of isotachioside (CD_3_OD; 600 MHz).

**
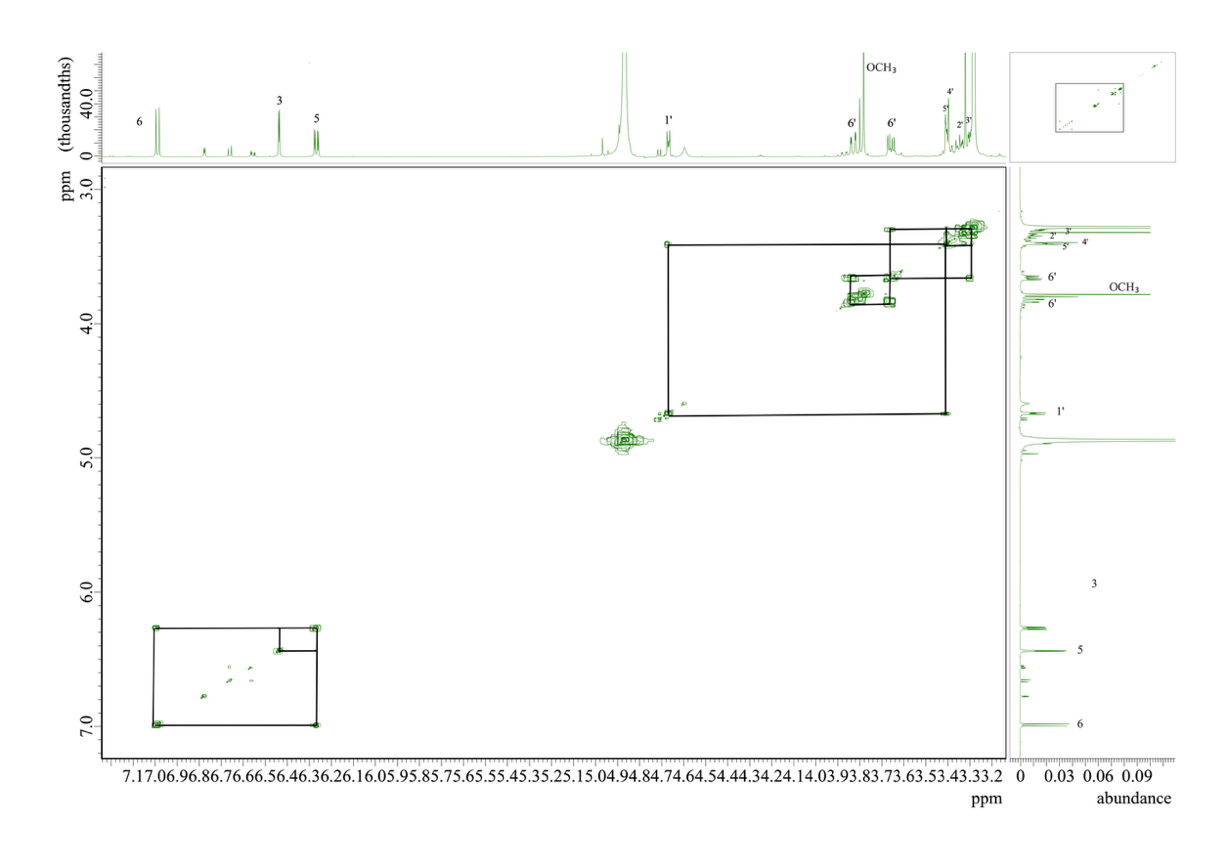
**

**Figure S4.** COSY spectrum of isotachioside.

**
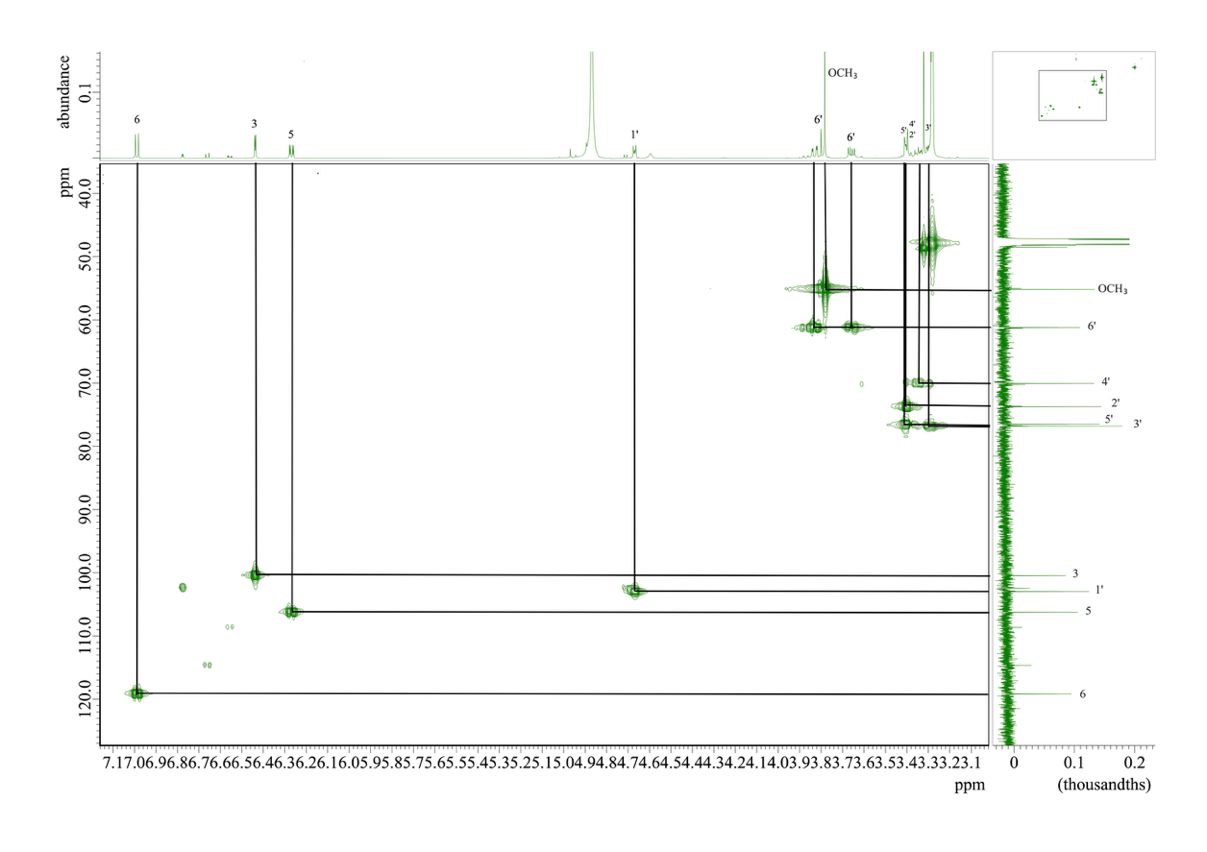
**

**Figure S5.** HMQC spectrum of isotachioside.

**
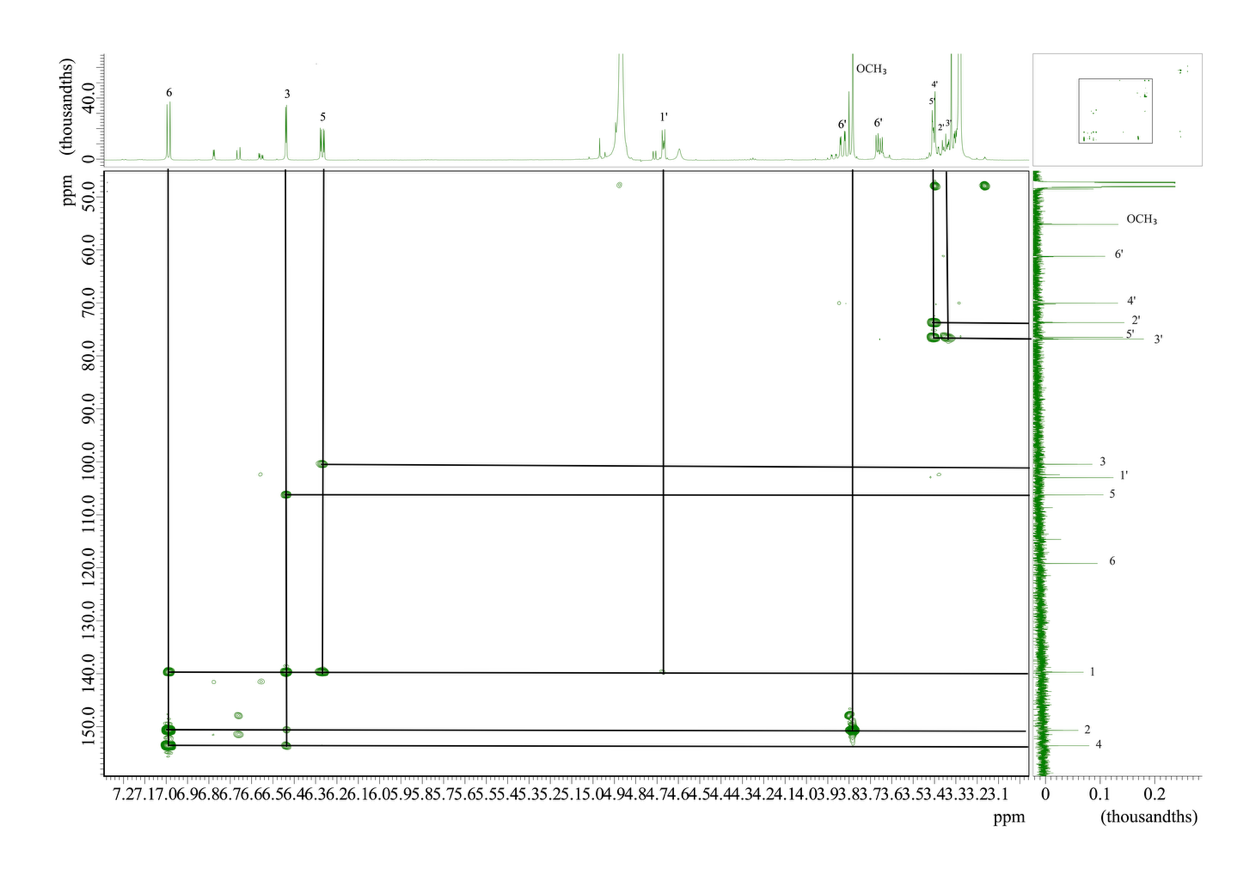
**

**Figure S6.** HMBC spectrum of isotachioside.

**Table S2.** ^13^C (CD_3_OD) data: aucubin (**2**), 10-hydroxymajoroside (**3**) and 10-acetoxymajoroside (**4**).

| **Positions** | **2** | **3** | **4** |
| --- | --- | --- | --- |
| Aglycon | | | |
| 1 | 98.0 | 92.4 | 92.1 |
| 3 | 141.9 | 152.3 | 152.2 |
| 4 | 106.0 | 114.0 | 113.9 |
| 5 | 46.6 | 37.3 | 37.3 |
| 6 | 83.1 | 41.8 | 41.6 |
| 7 | 130.5 | 77.4 | 77.5 |
| 8 | 148.3 | 143.0 | 139.1 |
| 9 | 48.2 | 136.0 | 138.2 |
| 10 | 61.7 | 57.7 | 59.3 |
| 11 | - | 169.3 | 169.1 |
| OCH_3_ | - | 51.9 | 52.0 |
| Glucose | | | |
| 1' | 100.2 | 100.5 | 100.3 |
| 2' | 75.2 | 75.0 | 75.0 |
| 3' | 78.2 | 78.3 | 78.3 |
| 4' | 71.9 | 71.8 | 71.8 |
| 5' | 78.6 | 78.6 | 78.7 |
| 6' | 63.0 | 63.0 | 63.0 |
| CH_3_OCO | - | - | 172.9 |
| CH_3_OCO |  |  | 21.0 |

**Table S3.** ^1^H NMR (CD_3_OD) data: chemical shift (δ, ppm) and coupling constant (*J*, Hz) for aucubin (**2**), 10-hydroxymajoroside (**3**) and 10-acetoxymajoroside (**4**).

| **Positions** | **2** | **3** | **4** |
| --- | --- | --- | --- |
| Aglycon | | | |
| 1 | 4.94 (1H, d, 7.2 Hz) | 6.41 (1H, s) | 6.34 (1H, s) |
| 3 | 6.30 (1H, dd, 1.8/6.0 Hz) | 7.37 (1H, d, 1.8 Hz) | 7.37 (1H, d, 1.8 Hz) |
| 4 | 5.08 (1H, dd, 3.6/6.0 Hz) | - | - |
| 5 | 2.64 (1H, m) | 3.84 (1H, t, 7.2 Hz) | 3.85 (1H, t, 6.6 Hz) |
| 6 | 4.43 (1H, m) | 1.71 (1H, m) | 1.66 (1H, m) |
|  |  | 2.44 (1H, dd, 6.6/13.8 Hz) | 2.44 (1H, dd, 6.6/13.8 Hz) |
| 7 | 5.75 (1H, brs) | 4.76 (1H, d, 6.6 Hz) | 4.72 (1H, d, 2.4 Hz) |
| 9 | 2.88 (1H, t, 7.2 Hz) | - | - |
| 10 | 4.34 (1H, d, 15.6 Hz) | 4.22 (1H, dd, 3.0/14.4 Hz) | 4.92 (1H, d, 13.2 Hz) |
|  | 4.15 (1H, d, 15.0 Hz) | 4.42 (1H, d, 12.0 Hz) | 4.71 (1H, d, 4.2 Hz) |
| OCH_3_ | - | 3.69 (3H, s) | 3.69 (3H, s) |
| Glucose | | | |
| 1' | 4.67 (1H, d, 8.4 Hz) | 4.68 (1H, d, 7.8 Hz) | 4.69 (1H, d, 8.4 Hz) |
| 2' | 3.20 (1H, t, 7.8 Hz) | 3.15 (1H, t, 8.4 Hz) | 3.16 (1H, t, 7.8 Hz) |
| 3' | 3.37 (1H, t, 8.4 Hz) | 3.35 (1H, d, 9.0 Hz) | 3.30-3.39^†^ (1H) |
| 4' | 3.27^†^ (1H) | 3.26^†^ (1H) | 3.30-3.39^†^ (1H) |
| 5' | 3.26^†^ (1H) | 3.28^†^ (1H) | 3.30-3.39^†^ (1H) |
| 6' | 3.63 (1H, dd, 5.4/12.0 Hz) | 3.66 (1H, dd, 5.4/12.0 Hz) | 3.67 (1H, dd 6.6/12.0) |
|  | 3.85 (1H, dd, 1.8/12.0 Hz) | 3.89 (1H, dd, 2.4/12.0 Hz) | 3.90 (1H, dd, 2.4/12.0 Hz) |
| CH_3_ | - | - | 2.06 (3H, s) |

†: The *J* value could not be calculated due to interference.


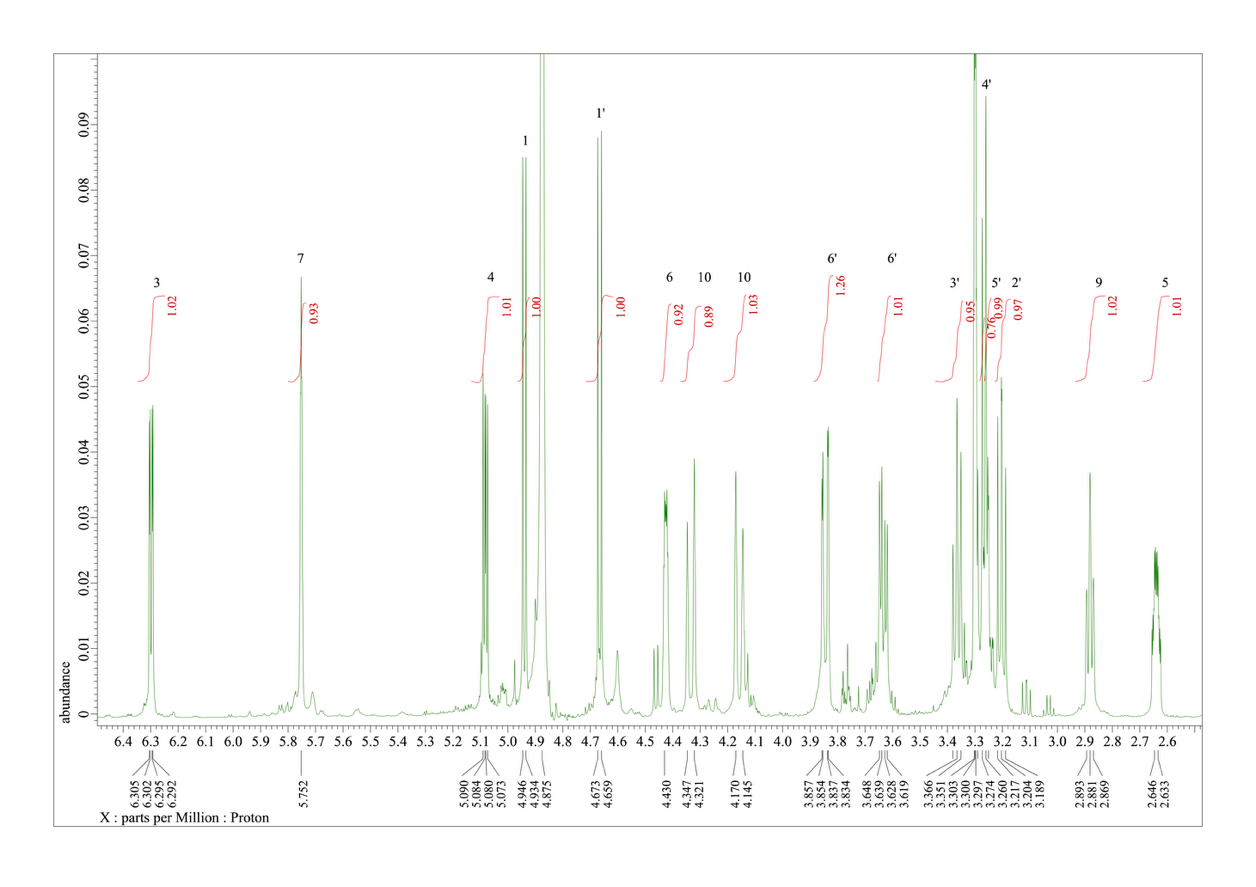


**Figure S7.** ^1^H-NMR spectrum of aucubin (CD_3_OD; 600 MHz).


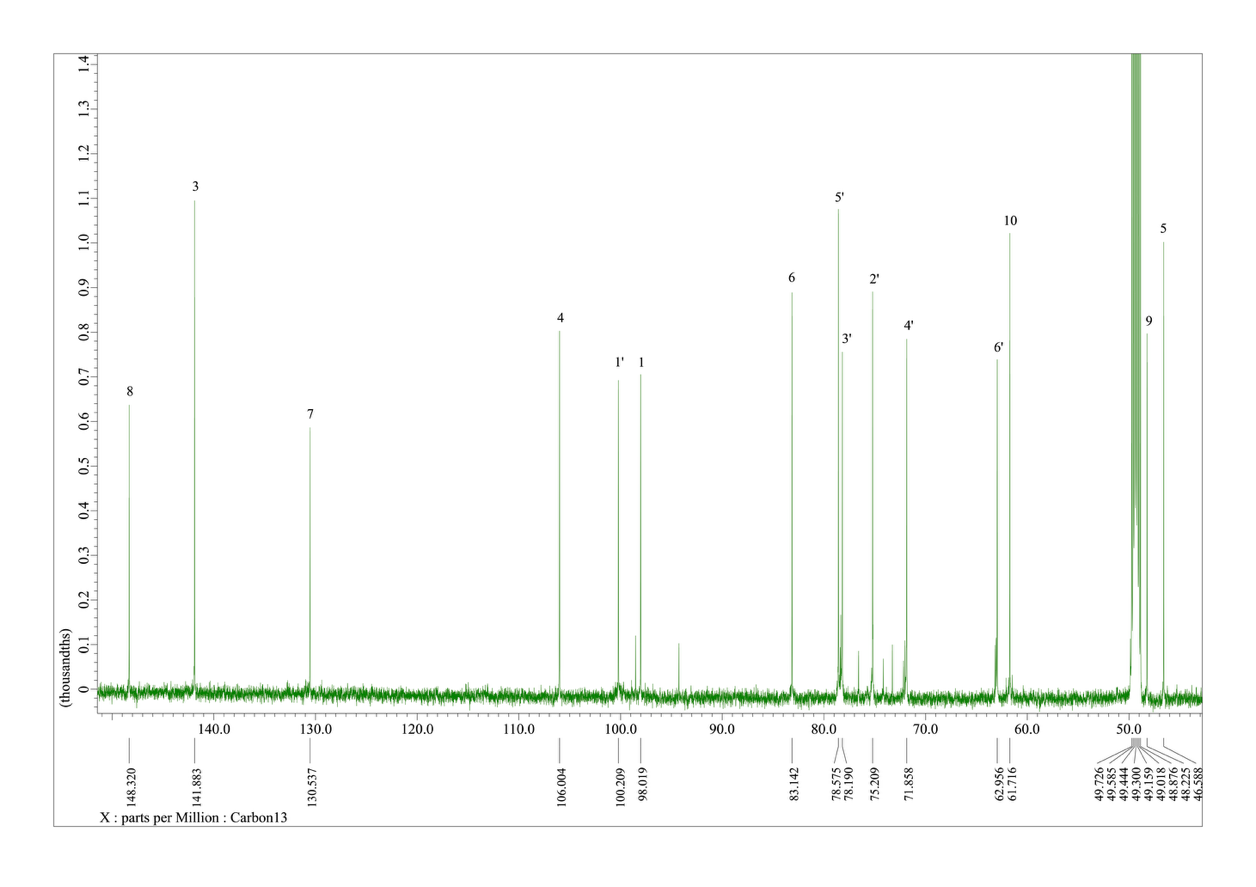


**Figure S8.** ^13^C-NMR spectrum of aucubin (CD_3_OD; 600 MHz).

**
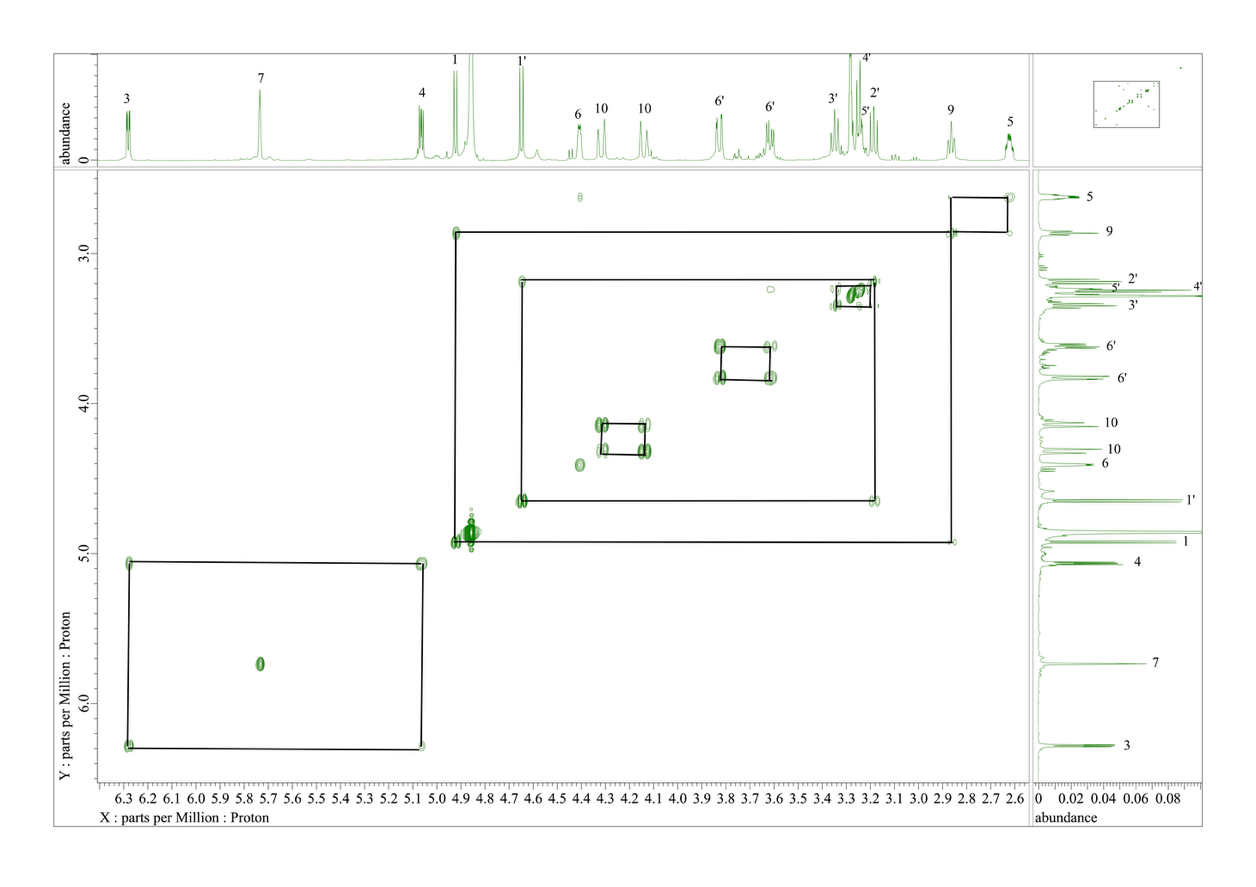
**

**Figure S9.** COSY spectrum of aucubin.


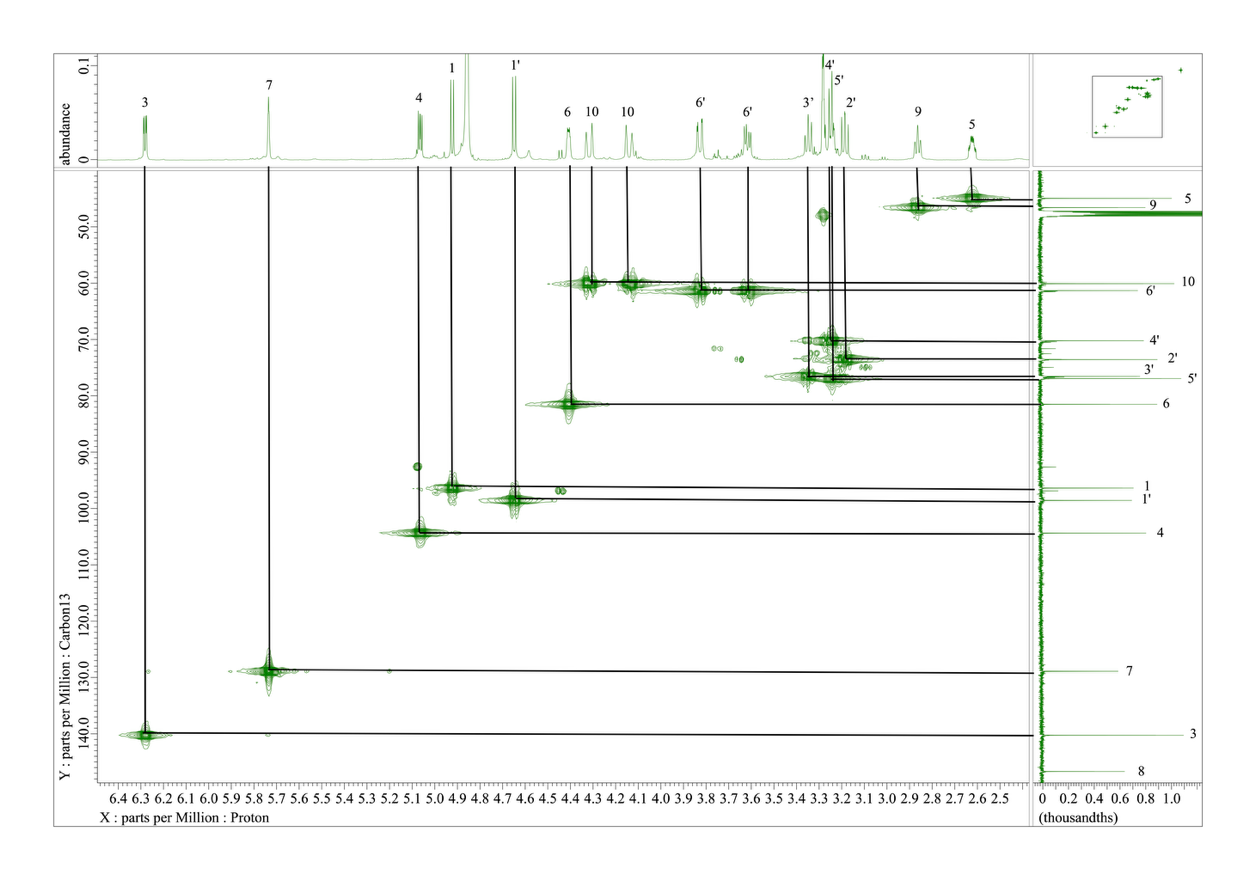


**Figure S10.** HMQC spectrum of aucubin.


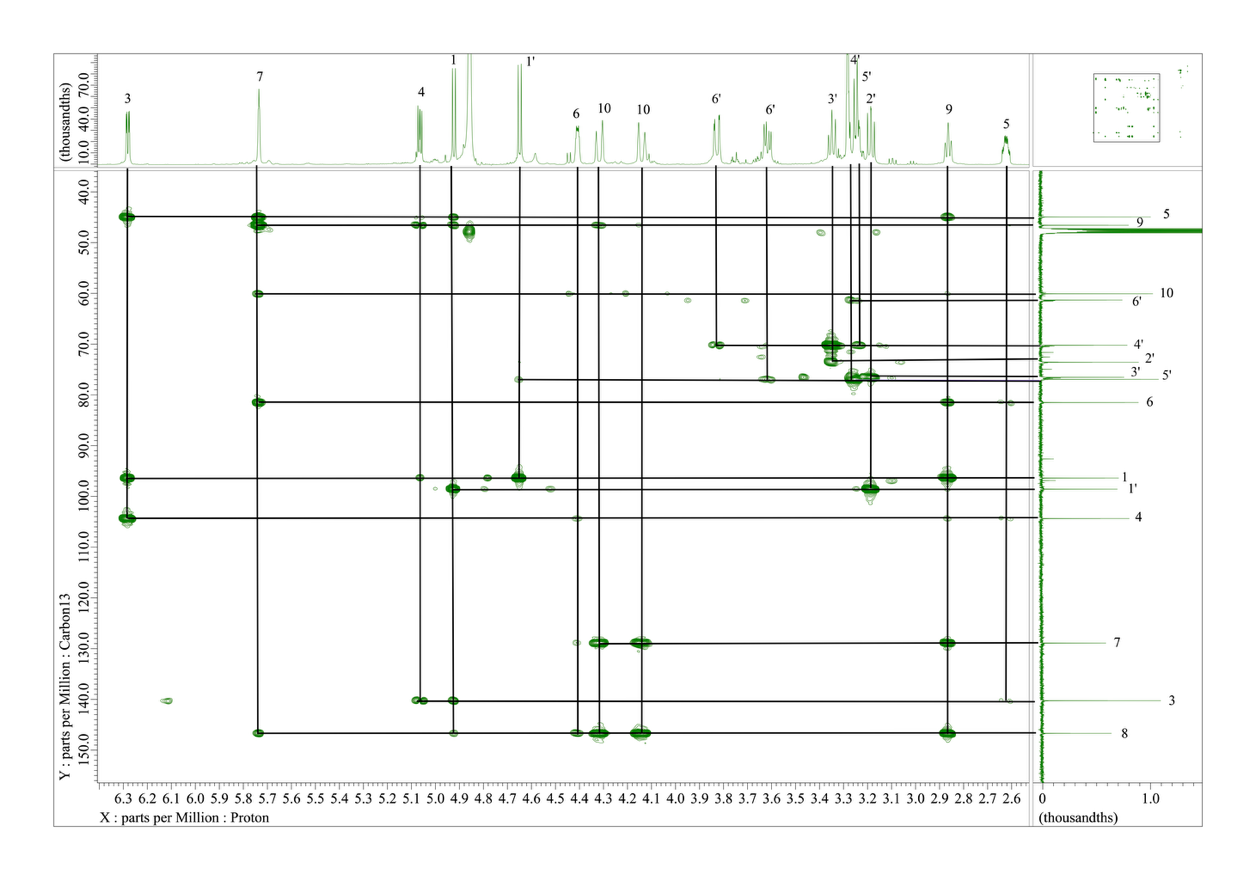


**Figure S11.** HMBC spectrum of aucubin.

**
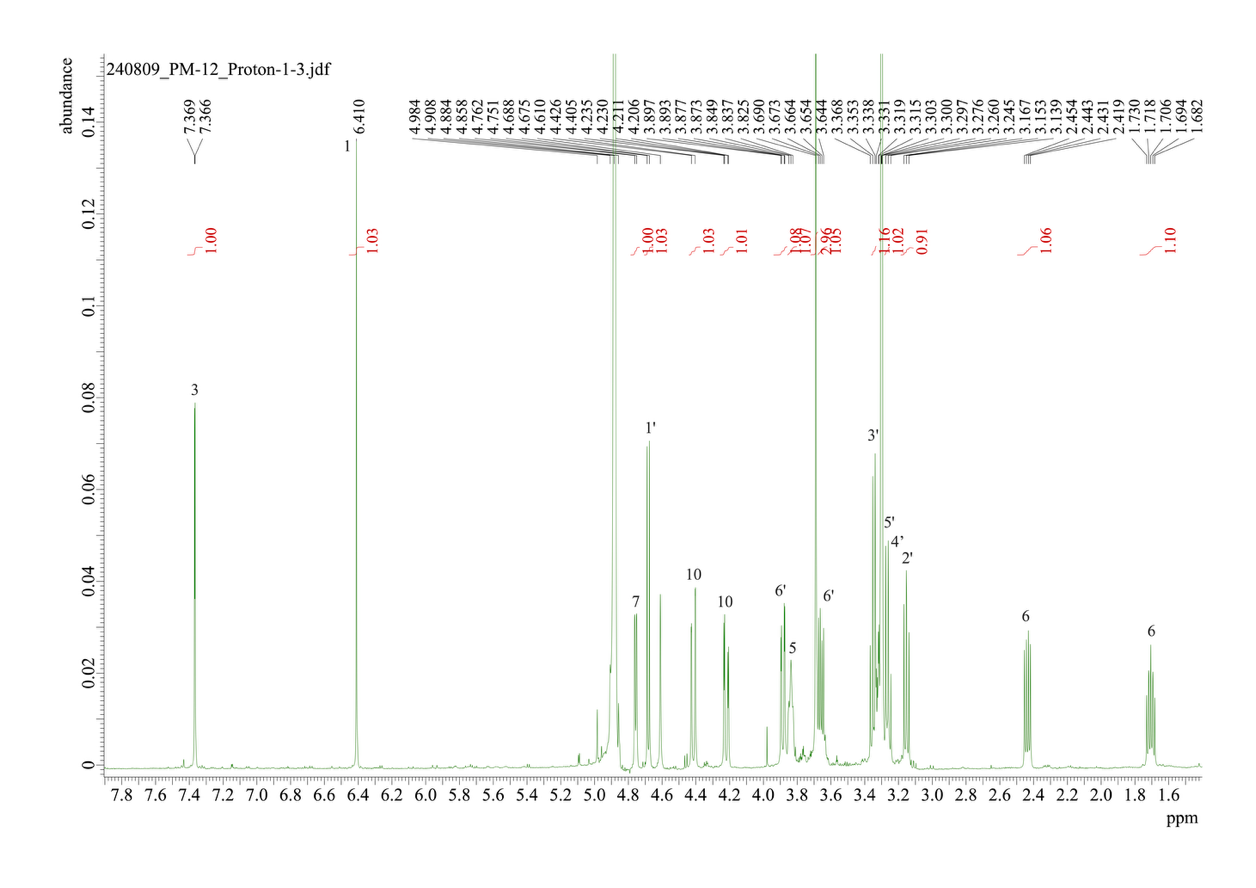
**

**Figure S12.** ^1^H-NMR spectrum of 10-hydroxymajoroside (CD_3_OD; 600 MHz).


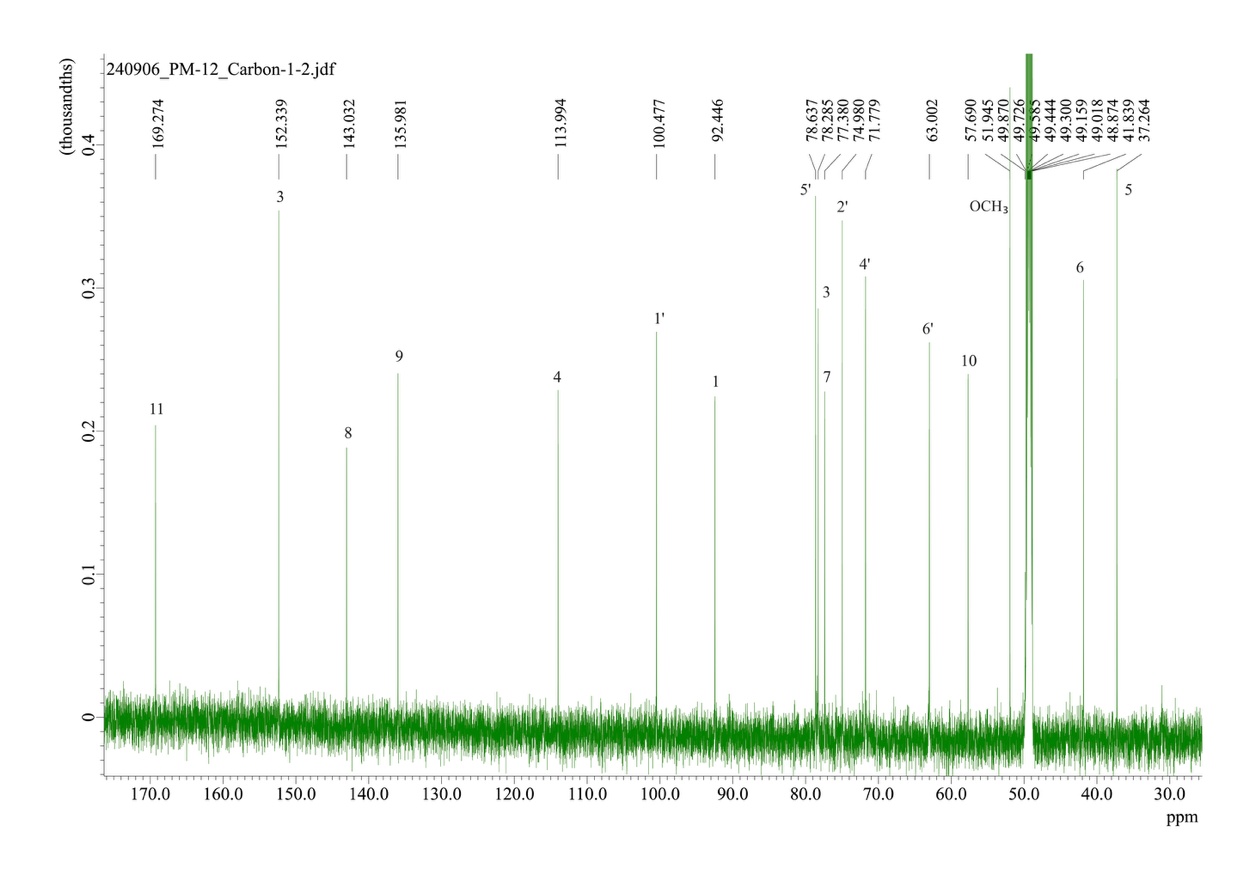


**Figure S13.** ^13^C-NMR spectrum of 10-hydroxymajoroside (CD_3_OD; 600 MHz).

**
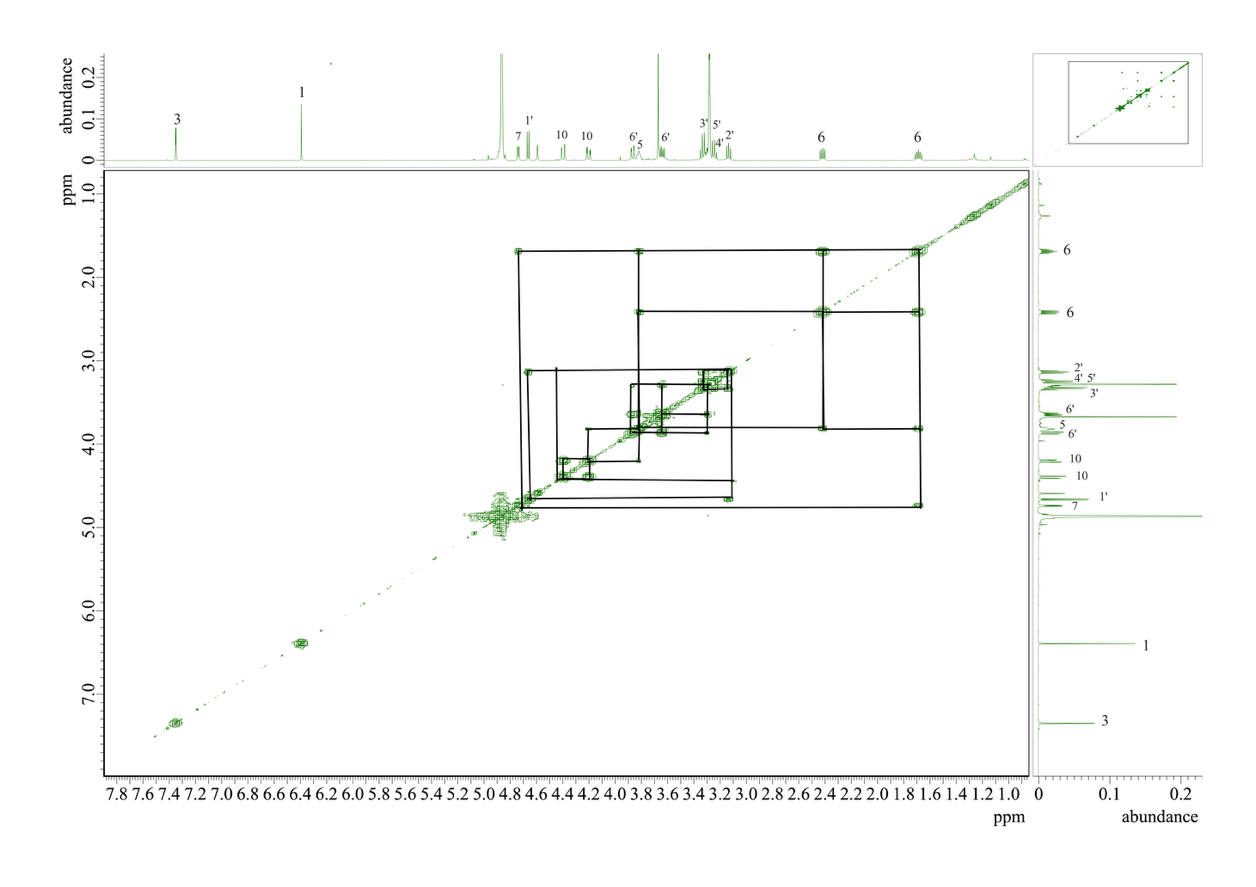
**

**Figure S14.** COSY spectrum of 10-hydroxymajoroside.

**
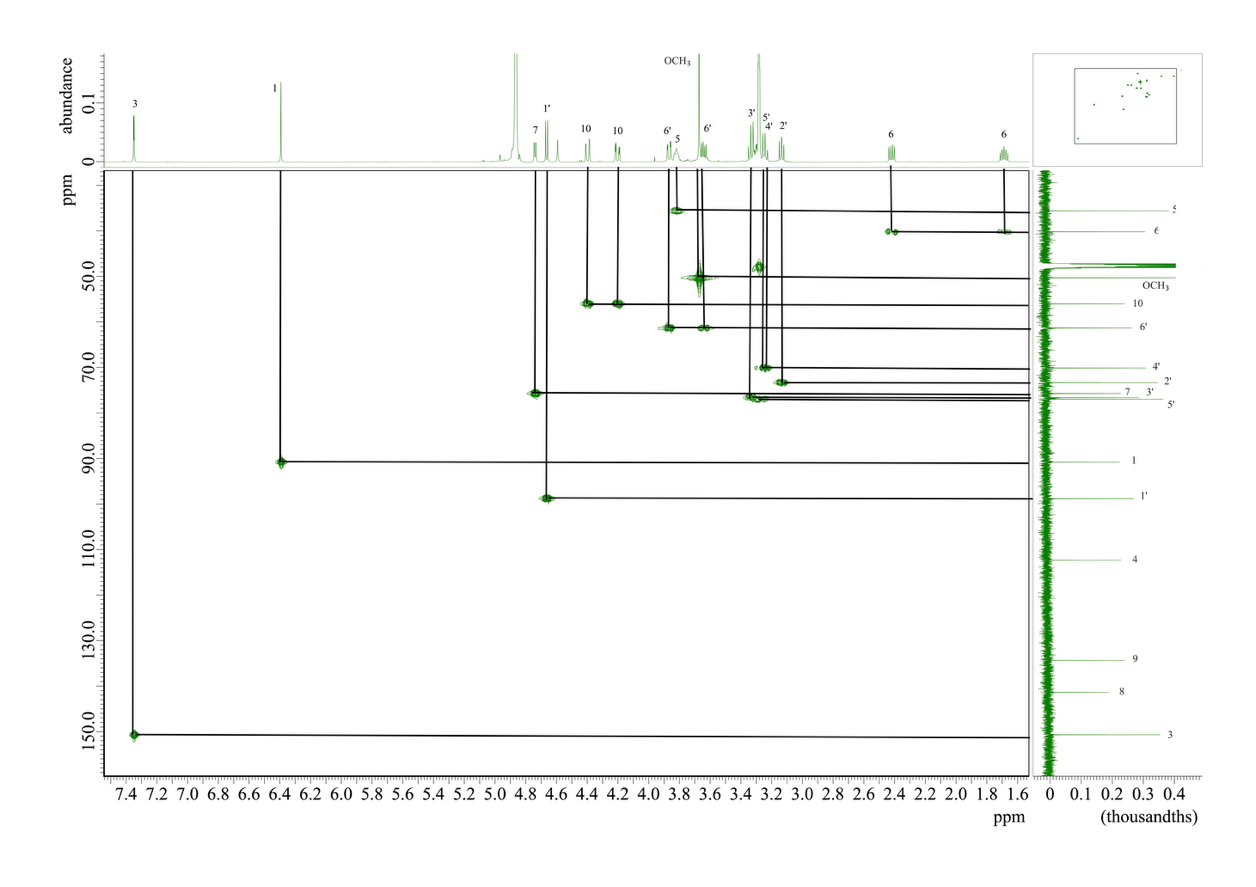
**

**Figure S15.** HMQC spectrum of 10-hydroxymajoroside.

**
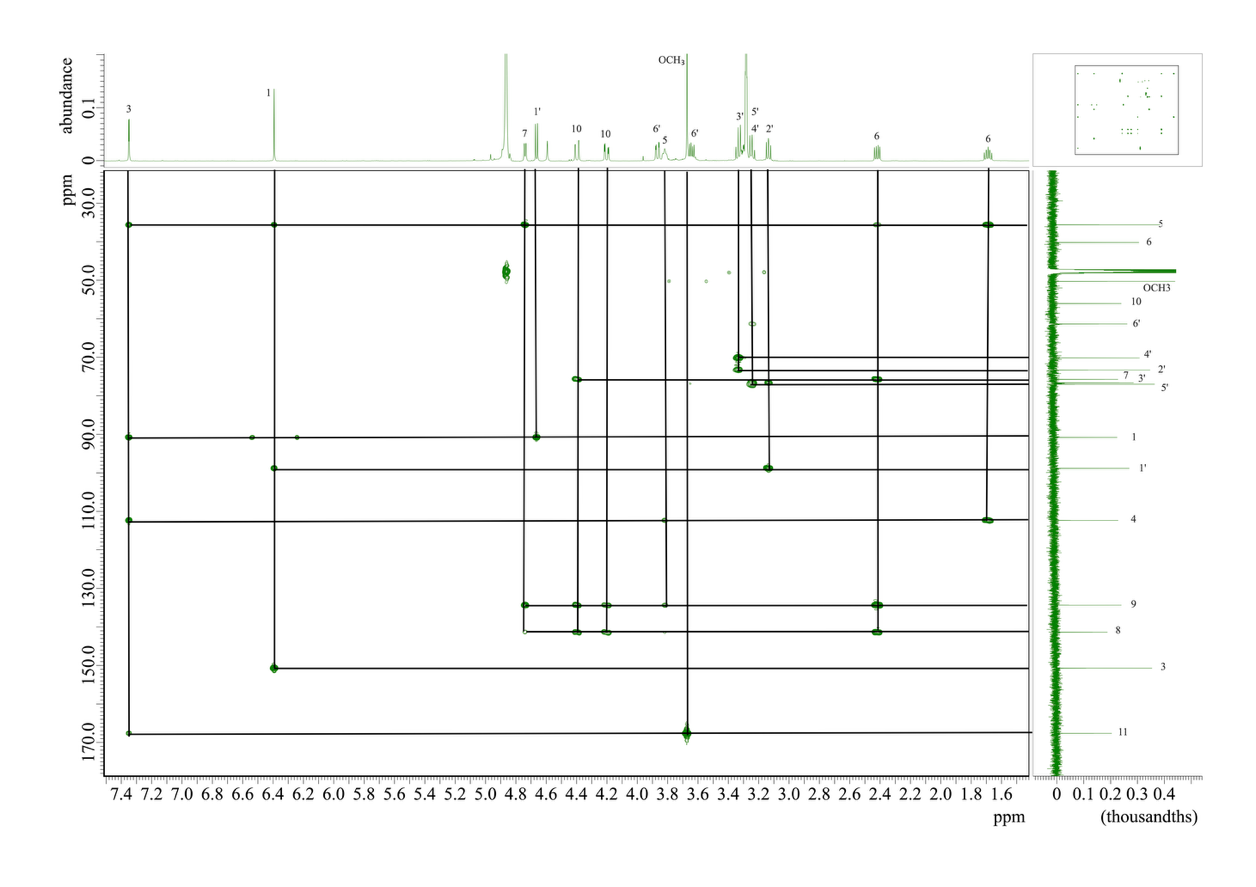
**

**Figure S16.** HMBC spectrum of 10-hydroxymajoroside.

**
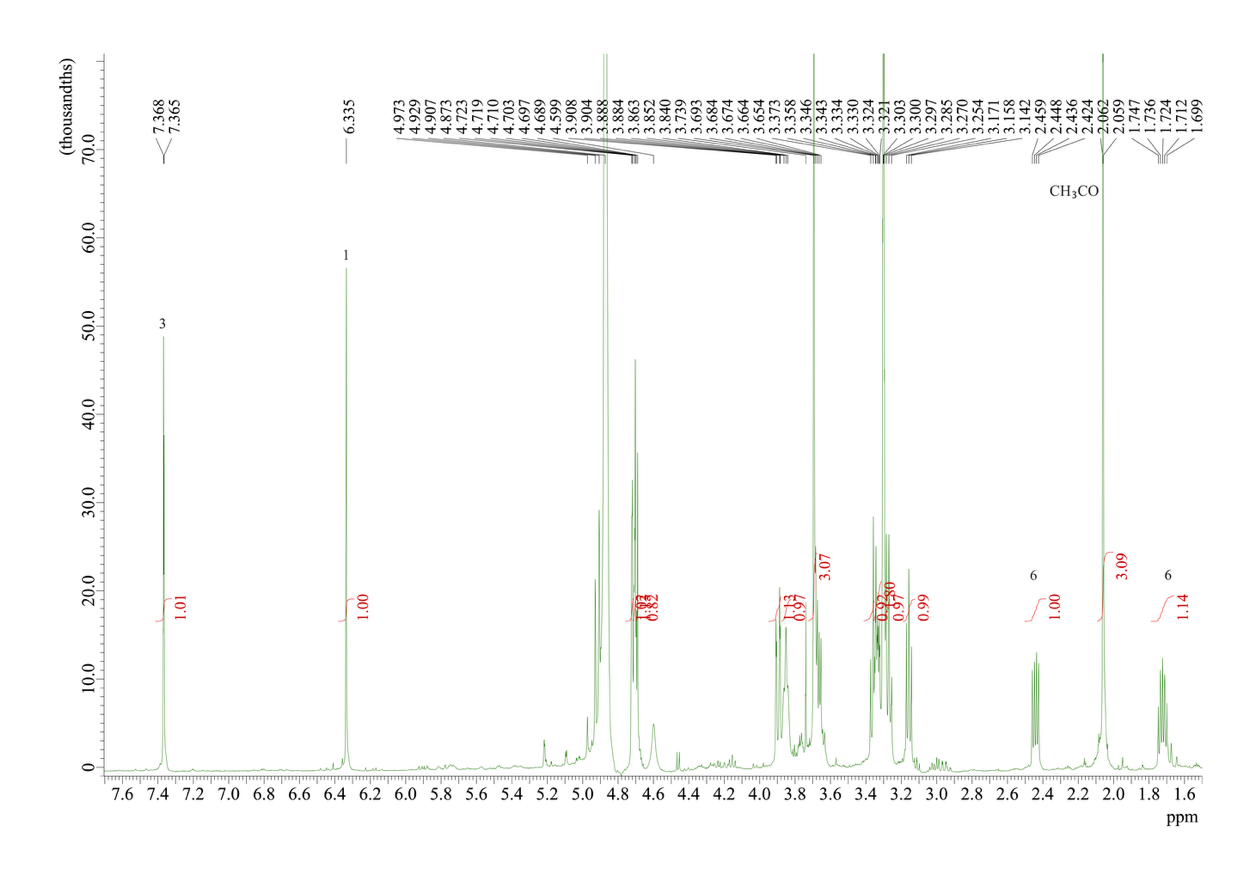
**

**Figure S17.** ^1^H-NMR spectrum of 10-acetoxymajoroside (CD_3_OD; 600 MHz).

**
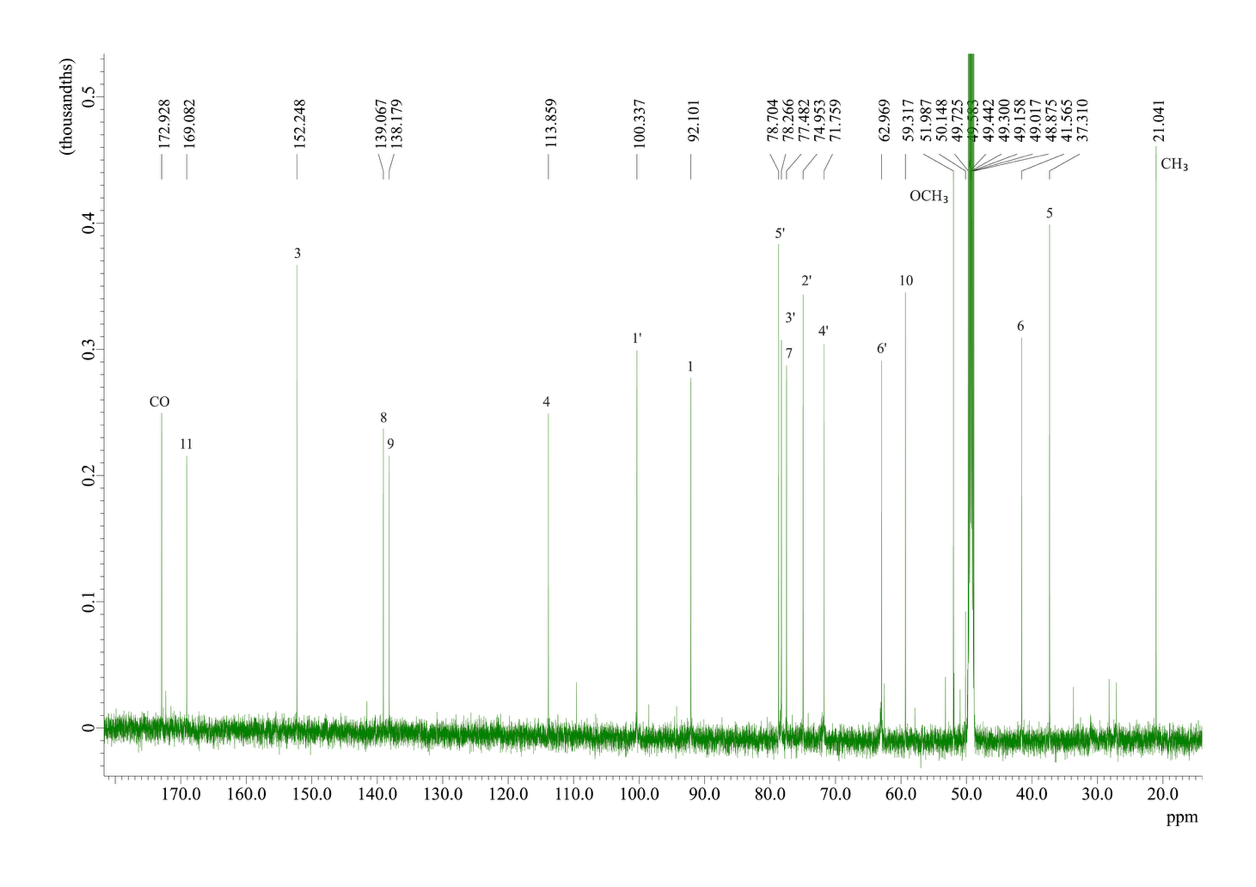
**

**Figure S18.** ^13^C-NMR spectrum of 10-acetoxymajoroside (CD_3_OD; 600 MHz).

**
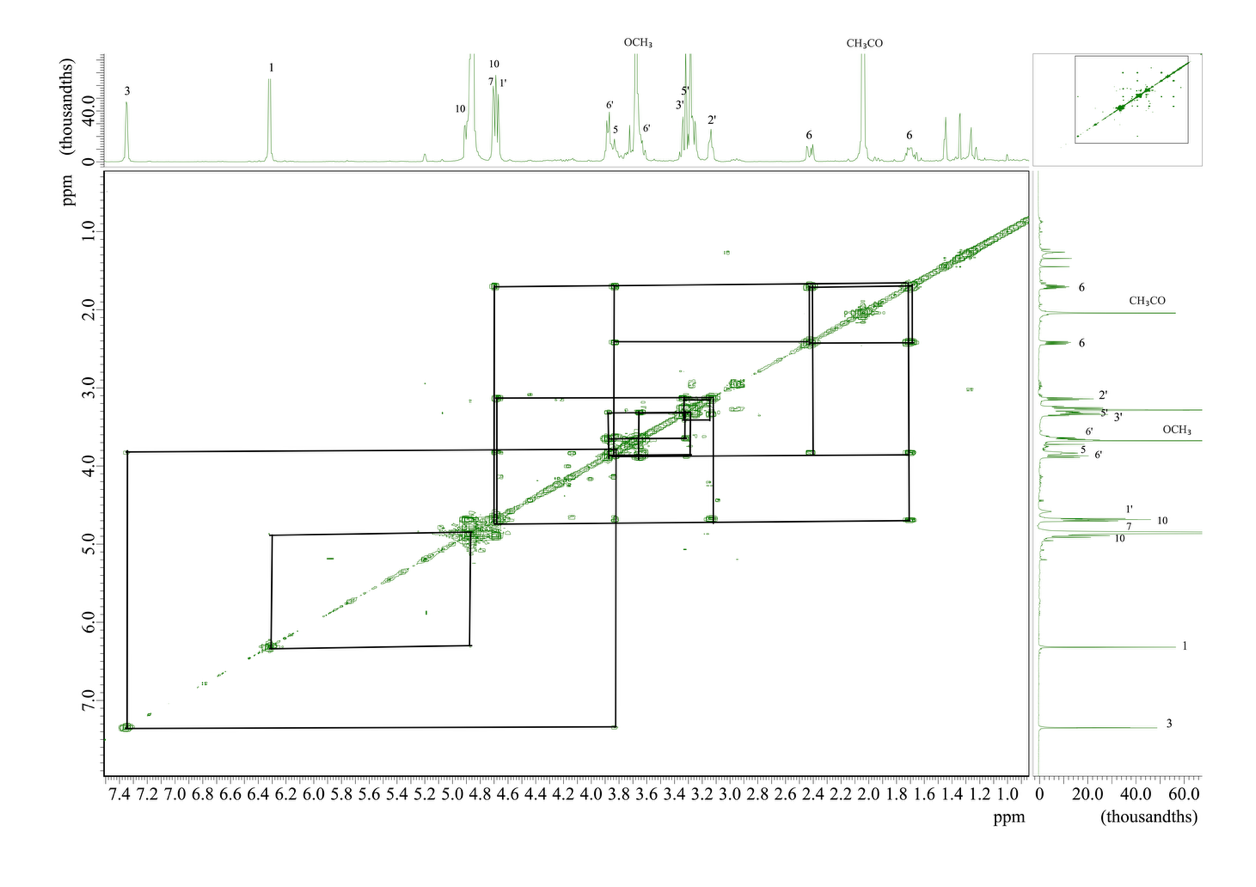
**

**Figure S19.** COSY spectrum of 10-acetoxymajoroside.

**
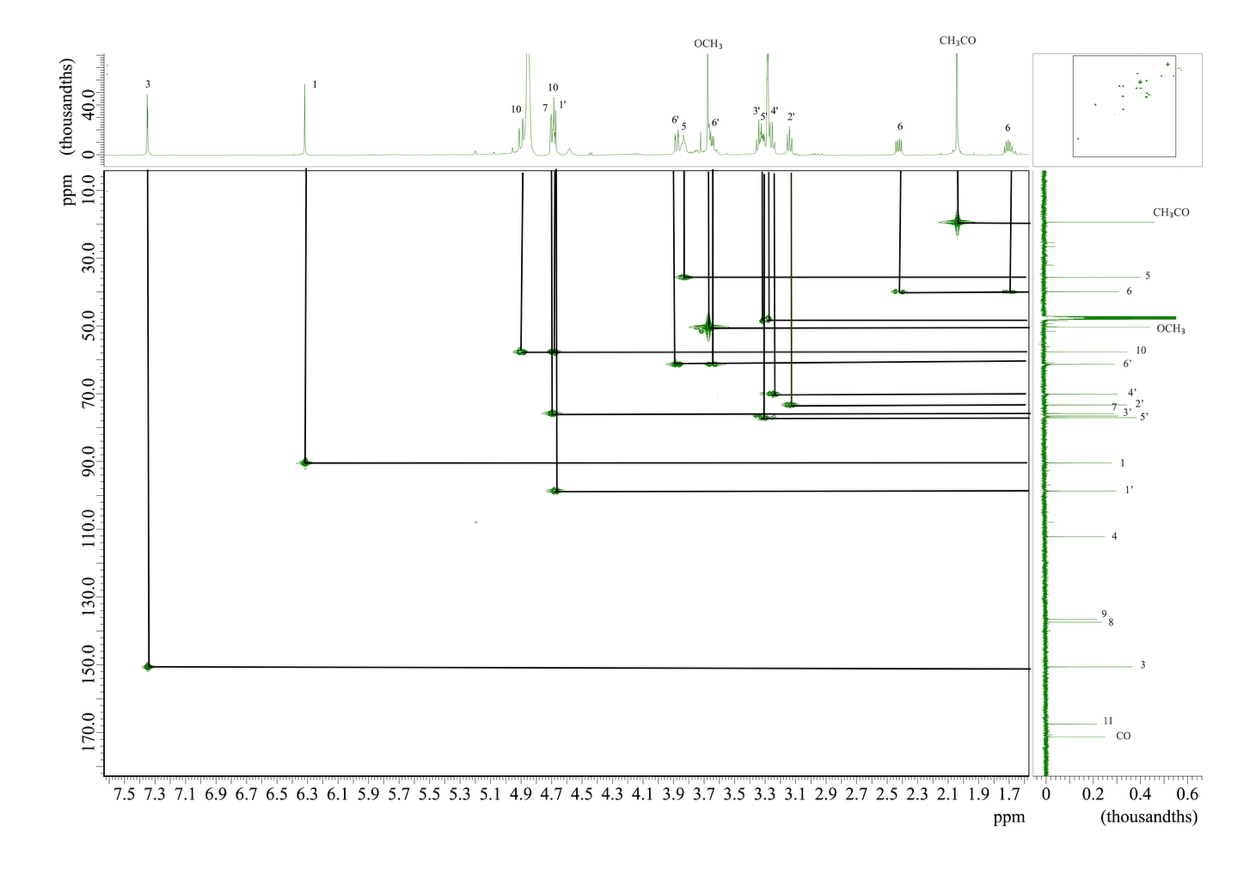
**

**Figure S20.** HMQC spectrum of 10-acetoxymajoroside.

**
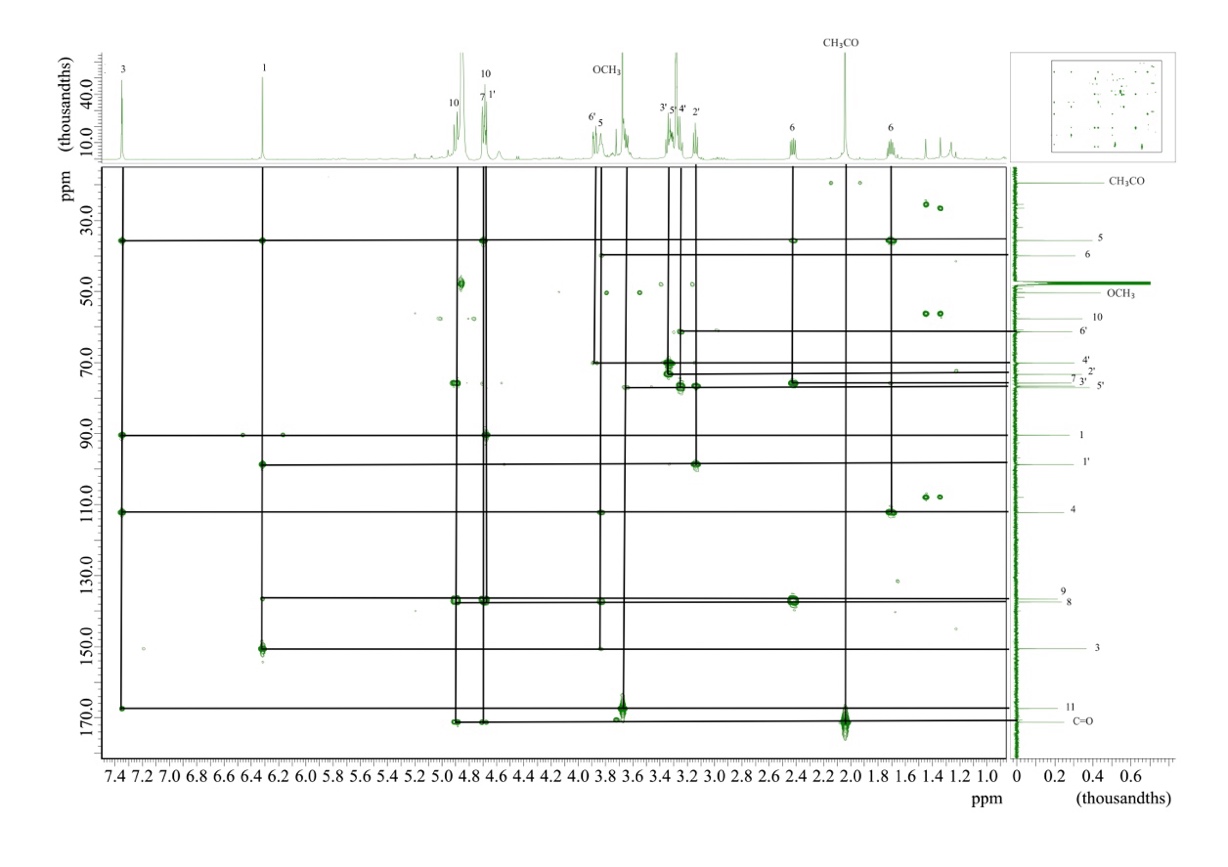
**

**Figure S21.** HMBC spectrum of 10- acetoxymajoroside.

**Table S4.** ^13^C NMR (CD_3_OD) data: acteoside (**6**)

| **C/H Atom** | **6** |
| --- | --- |
| 1 | 131.8 |
| 2 | 117.4 |
| 3 | 146.5 |
| 4 | 145.0 |
| 5 | 116.6 |
| 6 | 121.5 |
| *a* | 72.6 |
| *b* | 36.9 |
| Glucose |  |
| 1' | 104.5 |
| 2' | 76.5 |
| 3' | 81.9 |
| 4' | 70.8 |
| 5' | 76.3 |
| 6' | 62.7 |
| Rhamnose |  |
| 1'' | 103.3 |
| 2'' | 72.7 |
| 3'' | 72.4 |
| 4'' | 74.1 |
| 5'' | 70.7 |
| 6'' | 18.8 |
| Acyl moiety |  |
| 1''' | 128.0 |
| 2''' | 115.5 |
| 3''' | 147.2 |
| 4''' | 150.2 |
| 5''' | 116.8 |
| 6''' | 123.5 |
| *a*' | 115.0 |
| *b* ' | 148.3 |
| C=O | 168.6 |

**Table S5.** ^1^H NMR (CD_3_OD) data: chemical shift (δ, ppm) and coupling constant (*J*, Hz) for martynoside (**5**) and acteoside (**6**)

| **C/H Atom** | **5** | **6** |
| --- | --- | --- |
| 1 | - | - |
| 2 | 6.73 (1H, d, 2.4 Hz) | 6.68 (1H, d, 1.8 Hz) |
| 3 | - | - |
| 4 | - | - |
| 5 | 6.82 (1H, d, 7.8 Hz) | 6.66 (1H, d, 7.8 Hz) |
| 6 | 6.68 (1H, dd, 2.4/8.4 Hz) | 6.56 (1H, dd, 1.8/7.8 Hz) |
| *α* | 4.04 (1H, m) | 4.04 (1H, m) |
|  | 3.91 (1H, m) | 3.71 (1H, m) |
| *β* | 2.81 (1H, m) | 2.79 (2H, m) |
| OCH_3_ | 3.80 (3H, s) | - |
| Glucose |  |  |
| 1' | 4.38 (1H, d, 7.8 Hz) | 4.37 (1H, d, 7.8 Hz) |
| 2' | 3.38 (1H, brd, 9.6 Hz) | 3.38 (1H, brt, 9.0 Hz) |
| 3' | 3.78 -3.81^†^ | 3.80 (1H, t, 9.6 Hz) |
| 4' | 4.99 (1H, t, 9.6 Hz) | 4.98 (1H, t, 9.6 Hz) |
| 5' | 3.51-3.56^†^ | 3.50 (1H, d, 5.4 Hz) |
| 6' | 3.65^†^ | 3.61 (1H, dd, 4.8/13.8 Hz) |
|  | 3.53^†^ | 3.52 (1H, dd, 2.4/12.0 Hz) |
| Rhamnose |  |  |
| 1'' | 5.18 (1H, brs) | 5.18 (1H, d, 1.2 Hz) |
| 2'' | 3.98^†^ | 3.90 (1H, brt, 3.0 Hz) |
| 3'' | 3.57 (1H, dd, 3.6/9.6 Hz) | 3.56 (1H, dd, 3.6/9.6 Hz) |
| 4'' | 3.28 (1H, d, 9.6 Hz) | 3.27 (1H, d, 9.0 Hz) |
| 5'' | 3.51-3.56^†^ | 3.53-3.56^†^ |
| 6'' | 1.09 (1H, d, 6.0 Hz) | 1.08 (1H, d, 6.6 Hz) |
| Acyl moiety |  |  |
| 1'''' | - | - |
| 2'''' | 7.18 (1H, brs) | 7.04 (1H, d, 1.8 Hz) |
| 3'''' | - | - |
| 4'''' | - | - |
| 5'''' | 6.79 (1H, d, 7.8 Hz) | 6.76 (1H, d, 8.4 Hz) |
| 6'''' | 7.07 (1H, dd, 1.8/8.4 Hz) | 6.95 (1H, dd, 2.4/8.4 Hz) |
| *α* ' | 6.36 (1H, d, 16.0 Hz) | 6.26 (1H, d, 16.0 Hz) |
| *β* ' | 7.65 (1H, d, 16.0 Hz) | 7.58 (1H, d, 16.0 Hz) |
| C=O | - | - |
| OCH_3_ | 3.88 (3H, s) | - |

^†^: The *J* value could not be calculated due to interference.

**
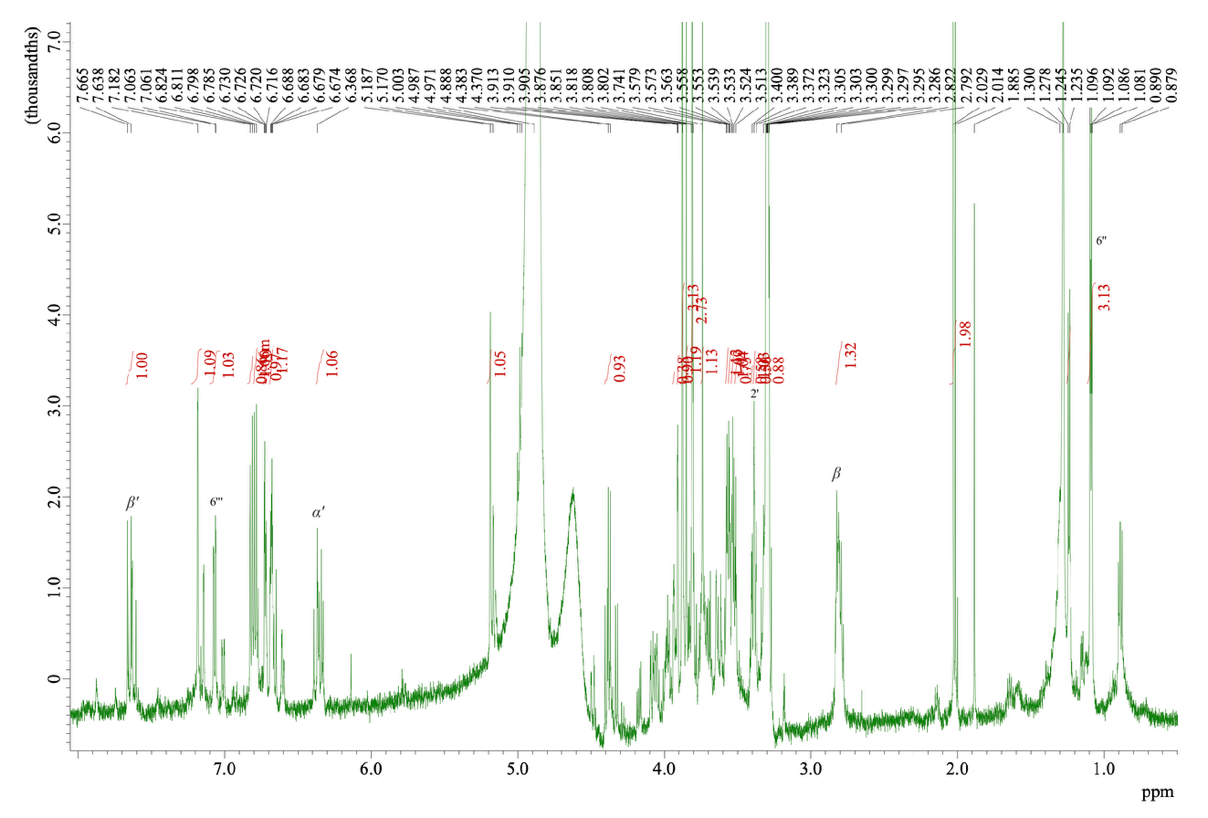
**

**Figure S22.** ^1^H-NMR spectrum of martynoside (CD_3_OD; 600 MHz).

**
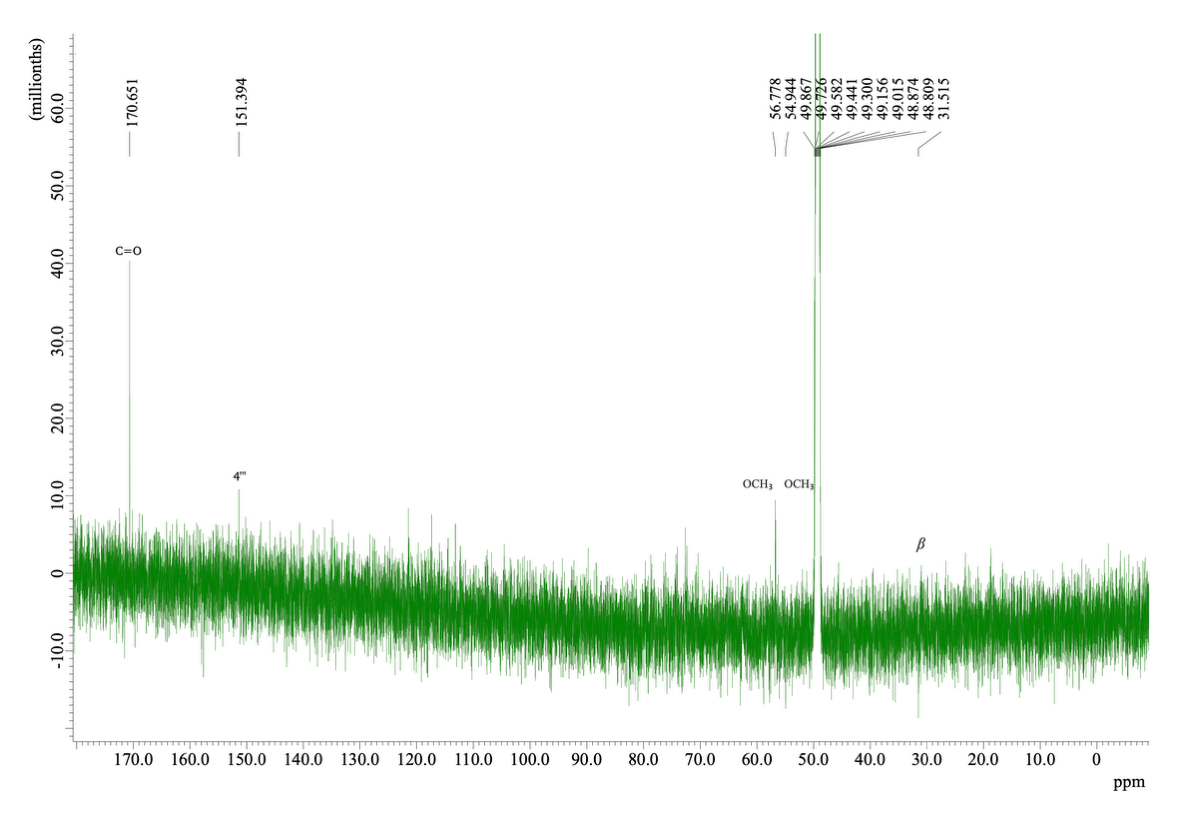
**

**Figure S23.** ^13^C-NMR spectrum of martynoside (CD_3_OD; 600 MHz).

**
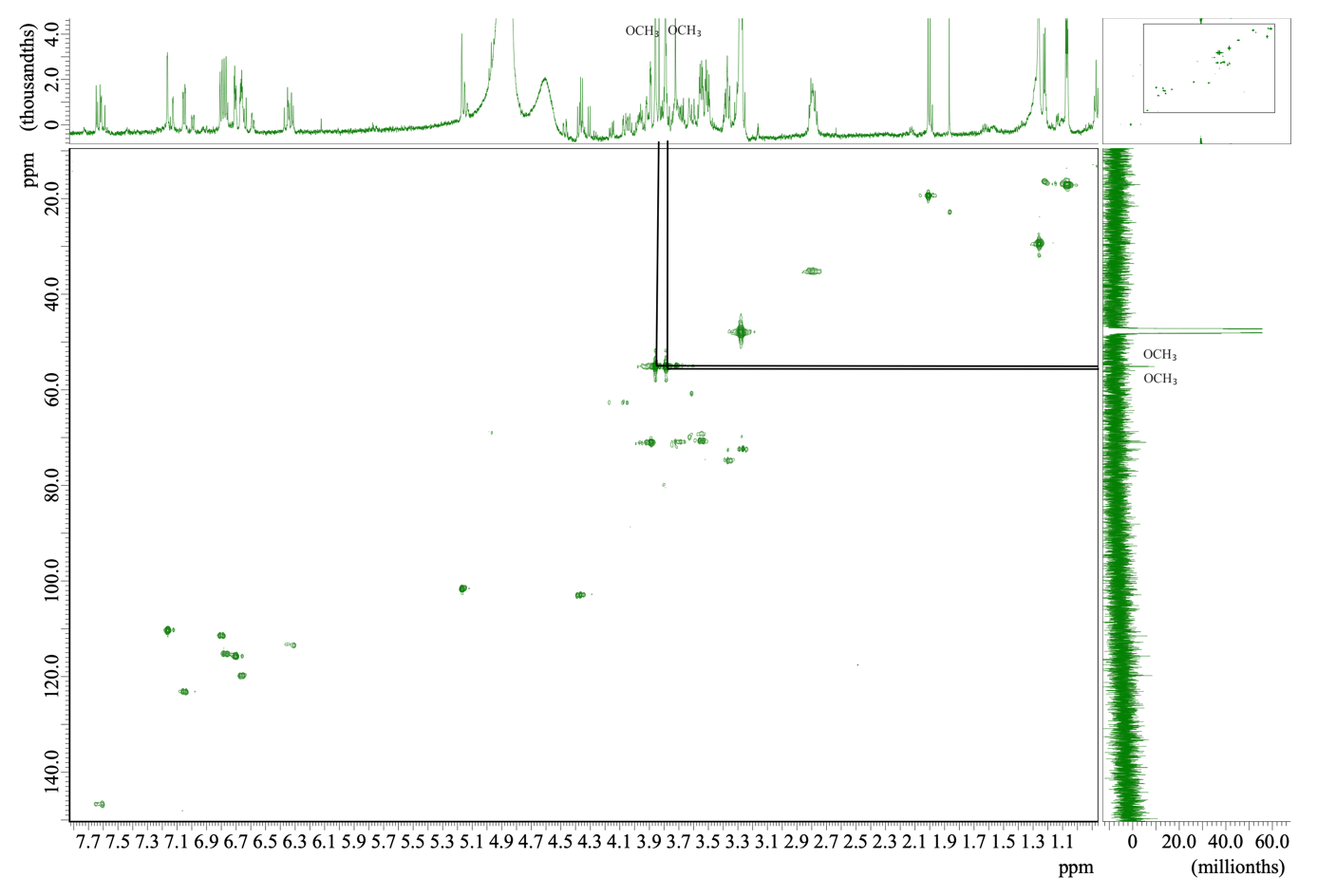
Figure S24.** HMQC spectrum of martynoside

**
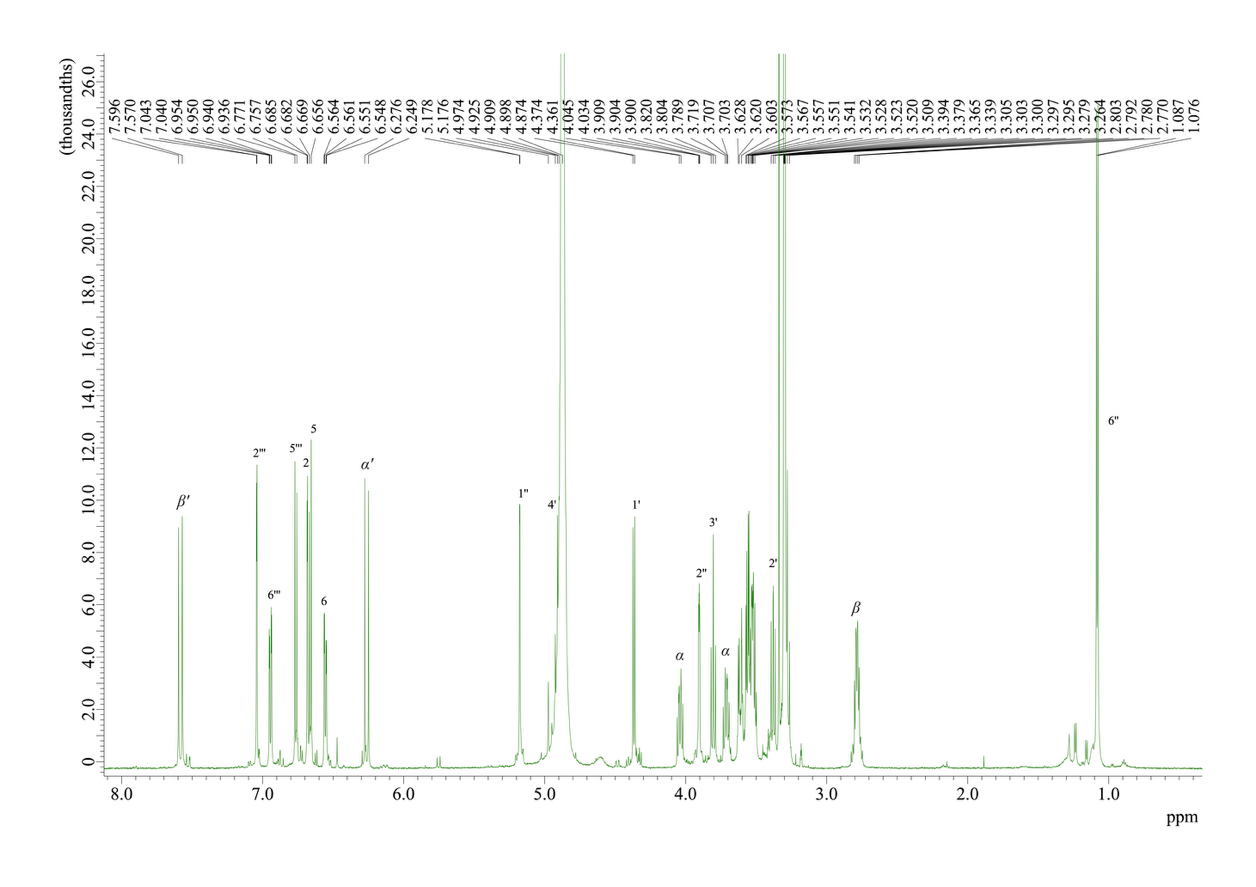
**

**Figure S25.** ^1^H-NMR spectrum of acteoside (CD_3_OD; 600 MHz).

**
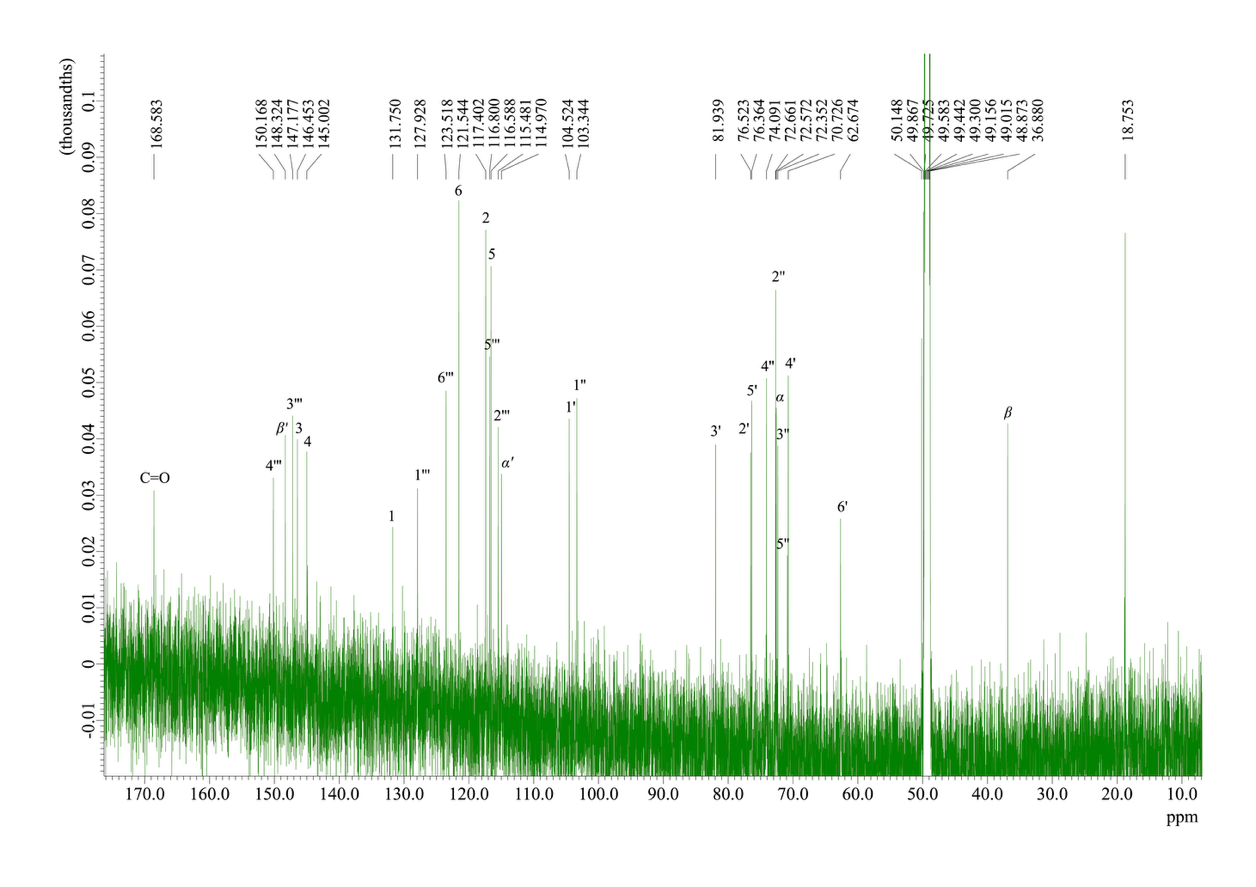
**

**Figure S26.** ^13^C-NMR spectrum of acteoside (CD_3_OD; 600 MHz).

**
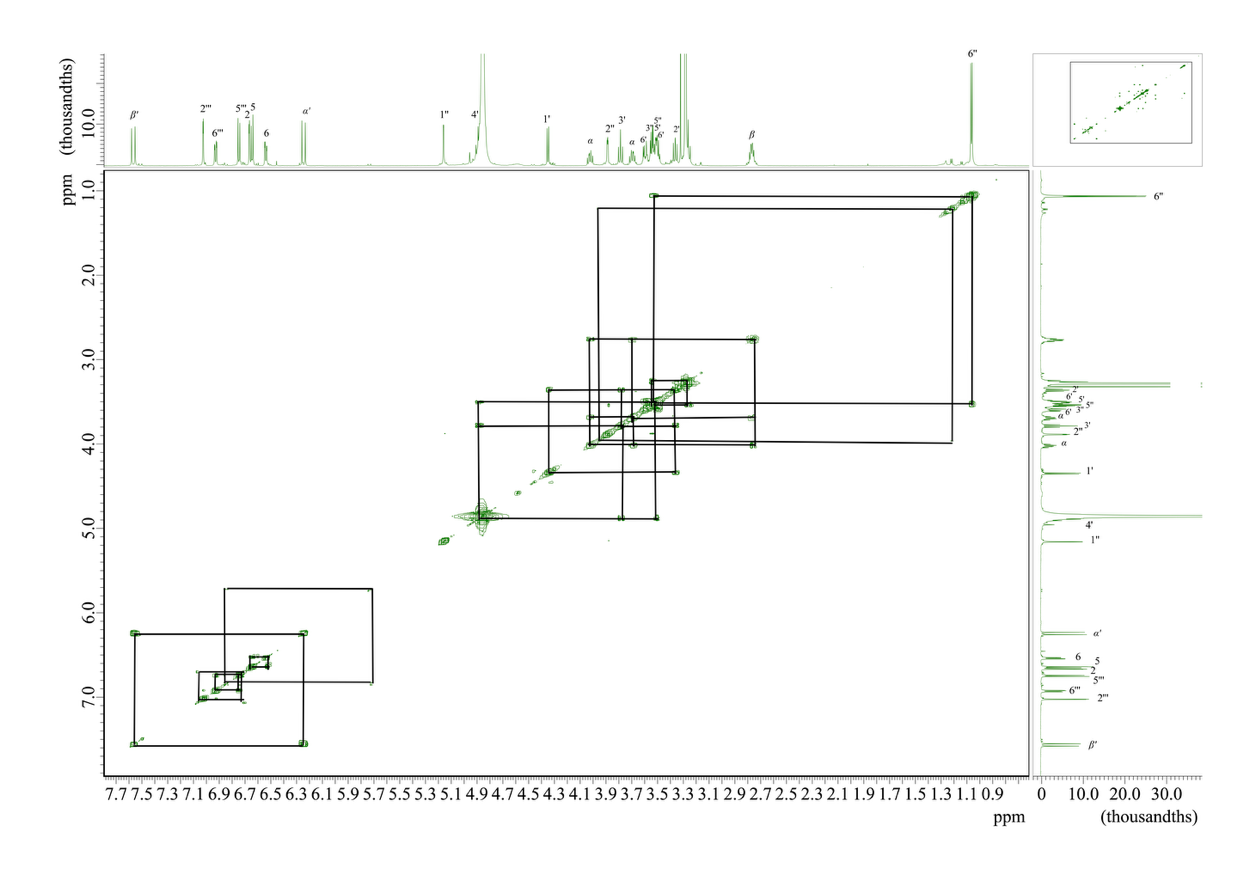
**

**Figure S27.** COSY spectrum of acteoside.

**
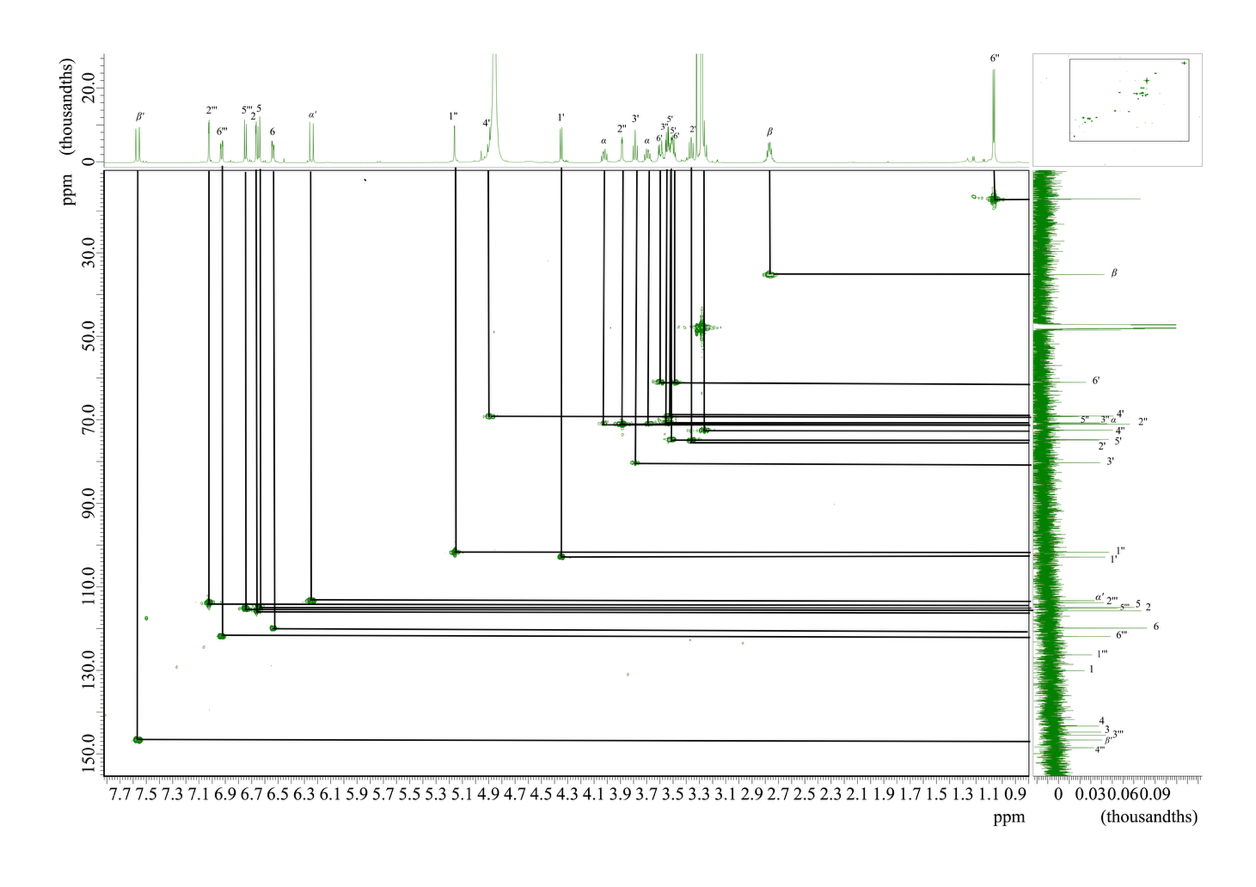
**

**Figure S28.** HMQC spectrum of acteoside.

**
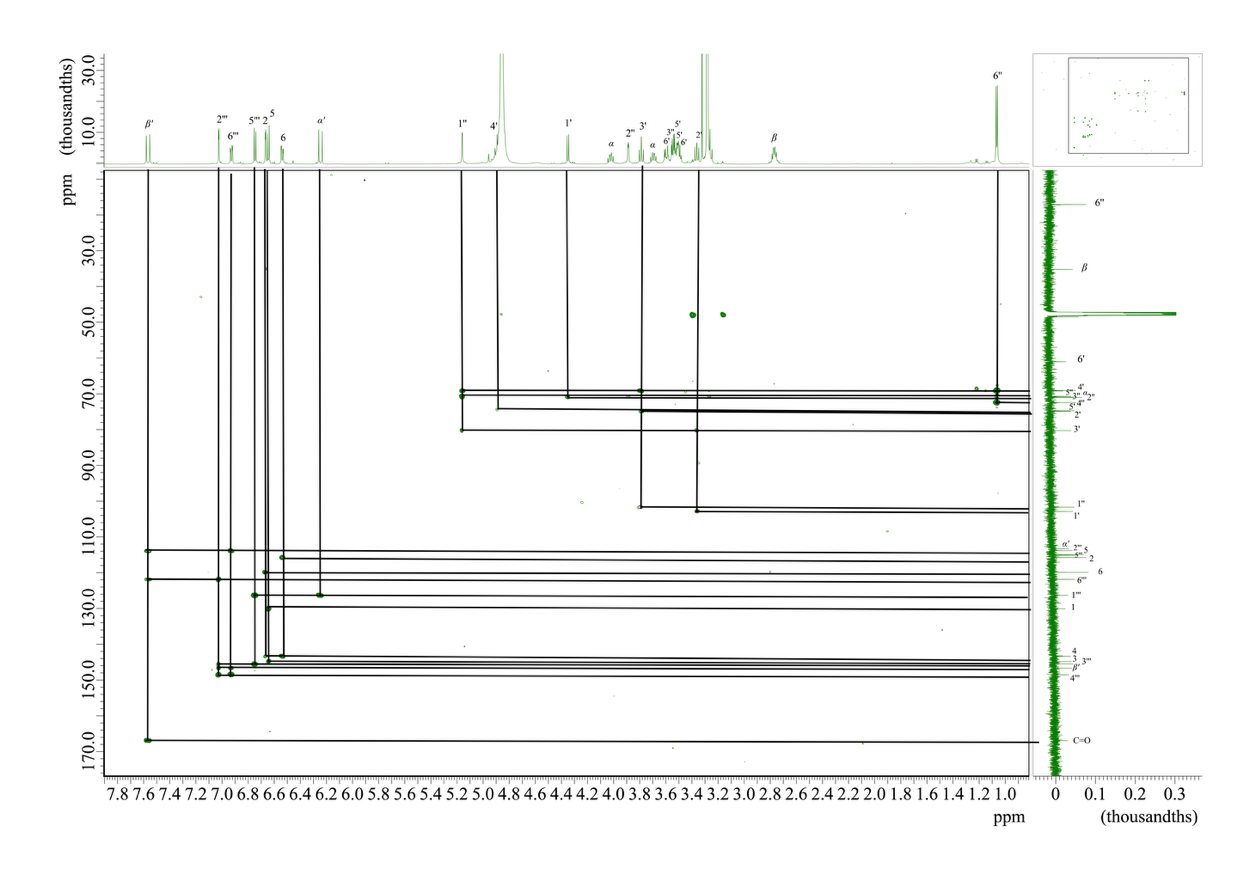
**

**Figure S29.** HMBC spectrum of acteoside.

**Table S6.** Effects of test and reference materials on wound contraction with circular excision model

| **Material** | **Extract type** | **Wound area ± S.E.M. (Contraction %)** | | | | | | |
| --- | --- | --- | --- | --- | --- | --- | --- | --- |
|  |  | **0** | **2** | **4** | **6** | **8** | **10** | **12** |
| Vehicle | - | 18.27 ± 2.13 | 18.34 ±2.90 | 16.25 ± 1.86 | 14.26 ± 1.93 | 8.21 ± 1.34 | 5.08 ± 1.23 | 4.22 ± 0.91 |
| Negative Control | - | 18.34 ± 2.19 | 19.07 ± 2.46 | 16.92 ± 2.11 | 13.67 ± 1.71 | 9.83 ± 1.62 | 7.21 ± 1.32 | 4.80 ± 1.16 |
| *Plantago major* subsp. *intermedia* | MeOH | 18.41 ± 2.11 | 18.31 ± 1.93 | 15.28 ± 2.14  (5.9) | 12.34 ± 1.03  (13.5) | 7.60 ± 1.15  (7.4) | 4.14 ± 0.63  (18.5) | 3.37 ±0.47  (20.1) |
|  | 80% EtOH | 18.16 ± 3.25 | 17.15 ±2.11  (6.5) | 14.64 ± 1.16  (9.9) | 11.95 ± 1.18  (16.2) | 7.24 ± 1.24  (11.8) | 4.09 ± 1.37  (19.4) | 3.23 ± 0.35  (23.4) |
|  | Water | 18.11 ± 2.37 | 18.44 ± 1.75 | 16.23 ± 1.88 | 15.61 ± 1.30 | 9.31 ± 1.88 | 5.15 ± 1.02 | 4.36 ± 0.61 |
| Madecassol^®^ |  | 18.34 ± 2.01 | 14.23 ± 2.34  (22.5) | 11.37 ± 2.02  **(30.0)*** | 7.16 ± 1.92  (**49.7)**** | 4.03 ± 0.97  (**50.9)**** | 1.18 ± 0.24  **(76.8)^***^** | 0.00 ± 0.00  **(100.00)^***^** |

Mean ± SEM; comparison with vehicle group and negative control; *: *p* <0.05; **: *p* <0.01; ***: *p* <0.001
